# Diet Quality and Dementia Risk in Older Adults With Alzheimer Pathology

**DOI:** 10.1001/jamanetworkopen.2026.20254

**Published:** 2026-06-25

**Authors:** Anja Mrhar, Adrián Carballo-Casla, Giulia Grande, Caterina Gregorio, Federico Triolo, Martina Valletta, Claudia Fredolini, Milica Gregorič Kramberger, Aleš Kuhar, Bengt Winblad, Laura Fratiglioni, Amaia Calderón-Larrañaga, Davide Liborio Vetrano

**Affiliations:** 1 Aging Research Center, Department of Neurobiology, Care Sciences and Society, Karolinska Institutet and Stockholm University, Stockholm, Sweden; 2 Biotechnical Faculty, University of Ljubljana, Ljubljana, Slovenia; 3 Center for Networked Biomedical Research in Epidemiology and Public Health, Madrid, Spain; 4 Stockholm Gerontology Research Center, Stockholm, Sweden; 5 Department of Protein Science, SciLifeLab, KTH Royal Institute of Technology, Solna, Sweden; 6 Affinity Proteomics Unit, SciLifeLab, KTH Royal Institute of Technology, Solna, Sweden; 7 Department of Neurology, University Medical Centre Ljubljana, Ljubljana, Slovenia; 8 Faculty of Medicine, University of Ljubljana, Ljubljana, Slovenia; 9 Department of Neurobiology, Care Sciences and Society, Division of Clinical Geriatrics, Karolinska Institutet, Huddinge, Sweden; 10 Department of Neurobiology, Care Sciences and Society, Division of Neurogeriatrics, Karolinska Institutet, Solna, Sweden; 11 Theme Inflammation and Aging, Karolinska University Hospital, Huddinge, Sweden

## Abstract

**Importance:**

Higher diet quality has been linked to reduced dementia incidence, but whether it buffers dementia onset in individuals with Alzheimer disease (AD) pathology or broader neurobiological risk is unclear.

**Objective:**

To explore the association of diet quality with dementia risk across biomarker levels of AD pathology (phosphorylated tau at threonine 217 [p-tau217]) and broader neurodegenerative and glial processes (neurofilament light chain [NFL], glial fibrillary acidic protein [GFAP]).

**Design, Setting, and Participants:**

This cohort study analyzed data from adults without dementia aged 60 years or older from the population-based Swedish National Study on Aging and Care in Kungsholmen, which enrolled participants between March 2001 and August 2004 and examined them up to 6 times until February 2016 to November 2019. Adherence to 3 dietary patterns was examined: the Alternate Mediterranean Diet (AMED), Alternative Healthy Eating Index (AHEI), and reversed Empirical Dietary Inflammatory Index (rEDII). Data were analyzed between September 2024 and August 2025 and reanalyzed in March and April 2026.

**Exposures:**

Baseline serum p-tau217, NFL, and GFAP concentrations and repeated adherence over 6 years to the AMED, AHEI, and rEDII dietary patterns.

**Main Outcomes and Measures:**

The primary outcome was all-cause dementia identified by clinical diagnosis, medical records, and death certificates. The secondary outcome was AD-related dementia. Dementia risk was analyzed using adjusted Cox regression models. Ten-year dementia probabilities and restricted mean time lost due to dementia were also estimated.

**Results:**

A total of 1865 participants were included (mean [SD] age at baseline, 70.5 [9.3] years; 1125 female [60.3%]). Over a mean follow-up of 8.4 years (range, <0.1 to 15.9 years), 240 participants developed dementia. Higher adherence to healthier dietary patterns was associated with lower dementia risk in participants with elevated AD and neurobiological risk biomarkers. For rEDII, each 1–*z*-score increase in adherence was associated with lower dementia risk among those with elevated p-tau217, NFL, and GFAP levels, with hazard ratios of 0.71 (95% CI, 0.58-0.88), 0.79 (95% CI, 0.66-0.95), and 0.73 (95% CI, 0.60-0.89), respectively. Associations of AMED and AHEI with lower dementia risk were generally found only among participants with lower biomarker levels. Similar findings were observed for AD-related dementia.

**Conclusions and Relevance:**

This cohort study of older adults found that adherence to a dietary pattern with lower inflammatory potential was associated with lower dementia risk among individuals with AD pathology and broader neurobiological risk. These findings reinforce the importance of targeted dietary dementia prevention strategies not only for the general population but also for individuals already at elevated risk.

## Key Points

**Question:**

Is higher diet quality associated with lower dementia risk among older adults with Alzheimer disease pathology or broader neurodegenerative and glial processes?

**Findings:**

In this cohort study of 1865 older adults without dementia followed up for up to 15 years, higher diet quality was associated with lower dementia risk. Among participants at higher risk, only a dietary pattern with lower inflammatory potential showed consistent inverse associations.

**Meaning:**

These findings reinforce the importance of targeted dietary dementia prevention strategies for the general population and individuals already at elevated risk.

## Teaser

This cohort study examines the association of diet quality with dementia risk across biomarkers for Alzheimer disease pathology and neurodegenerative and glial processes in older adults without dementia in Sweden.

## Introduction

Given the growing prevalence of dementia and limited availability of disease-modifying treatments, a greater understanding of modifiable risk factors is needed.^1,2^ Current research on nutrition has yielded promising, but not yet definitive, findings on how nutrients, foods, and dietary patterns may mitigate Alzheimer disease (AD), one of the leading causes of dementia, and modify dementia onset.^3^

The accumulation of AD pathology is necessary but not always sufficient for the onset of dementia.^4^ A growing body of evidence has shown that several blood-based biomarkers are correlated with cerebrospinal fluid and cerebral positron emission tomography biomarkers of AD pathology and may hence be used to predict all-cause and AD-specific dementia.^5,6^ Among these blood-based biomarkers, phosphorylated tau at threonine 217 (p-tau217) is associated with amyloid and tau positivity and is one of the most accurate blood-based biomarkers for early detection of AD.^6^ In addition, nonspecific biomarkers, such as neurofilament light chain (NFL), a marker of neuronal injury, and glial fibrillary acidic protein (GFAP), indicative of astrocyte activation or damage, have been shown to predict dementia across various neurodegenerative conditions, including AD.^7,8^ Taken together, these blood-based biomarkers could be used to identify persons at risk of dementia who may benefit from specific preventive recommendations, including diet.^8,9^

In this regard, epidemiologic studies have been shifting focus from single-nutrient assessments to dietary patterns, which better capture the complex interrelationships between food components and their associations with brain pathology, cognitive decline, and dementia incidence.^10^ The Mediterranean diet is the most extensively examined pattern, albeit not the only one, with respect to cognitive outcomes and risk of dementia.^3^ Evidence from observational studies and randomized clinical trials also has indicated an association between the Mediterranean diet and other healthy dietary patterns and better cardiovascular health (eg, diabetes, hypertension, cardiovascular disease),^11^ which has an important role in brain health and dementia development.^12^ This association suggests a potential favorable role of healthy dietary patterns, such as the Alternative Healthy Eating Index (AHEI) that is based on the main nutrients and food items linked to cardiovascular health.^13,14^ In contrast, dietary patterns characterized by a high inflammatory potential, such as dietary inflammatory indices, have been associated with worse brain health markers^15,16^ and a higher risk of dementia.^17,18,19^

Although several studies of dementia have assessed the association of adherence to dietary patterns with neuroimaging and cerebrospinal fluid AD biomarkers, to our knowledge, no study has analyzed how dietary patterns may modify dementia risk across strata of biological risk, including among individuals with AD pathology. With this aim, we performed a longitudinal, population-based study among older adults (aged ≥60 years) without dementia with different levels of biomarker-defined dementia risk. To allow for side-by-side comparisons across healthy diets, we analyzed in parallel 3 dietary patterns consistently associated with cognitive decline and brain health: the Alternate Mediterranean Diet (AMED), AHEI, and Empirical Dietary Inflammatory Index (EDII).

## Methods

### Study Design and Population

This cohort study used data from the population-based Swedish National Study on Aging and Care in Kungsholmen (SNAC-K), an ongoing cohort study that recruited participants aged 60 years or older between March 2001 and August 2004 (participation rate, 73%), and followed up with them every 3 years (participants aged ≥78 years) or 6 years (participants aged <78 years) through February 2016 to November 2019. We included baseline biomarker measurements; repeated diet and covariate assessments from baseline, 3-year, and 6-year visits; and dementia ascertainment over 15 years. The protocols of the SNAC-K study were approved by the Regional Ethical Review Board in Stockholm. All participants or their proxies provided written informed consent for participation and the use of their data in research. The study followed the Strengthening the Reporting of Observational Studies in Epidemiology (STROBE) reporting guideline for cohort studies.^20^

The SNAC-K protocol is described elsewhere.^21^ The detailed study design is described in eMethods 1 in Supplement 1.

### Study Variables and Data Collection

#### Diet

Data from a validated 98-item food frequency questionnaire were used to evaluate habitual diet.^22^ Adherence to the AMED, scored according to Fung et al^23^; AHEI, following the procedure by Chiuve et al^24^; and the reverse EDII (rEDII) proposed by Tabung et al^25^ was examined. Higher dietary pattern adherence scores indicated higher diet quality. Detailed descriptions of dietary pattern adherence estimation are provided in eMethods 2 in Supplement 1, and information on the components and scoring of each dietary pattern is presented in eTables 2 to 4 in Supplement 1.

#### Blood-Based Biomarkers of AD and Neurobiological Risk

Baseline data on blood-based biomarkers reflecting AD-related pathology (p-tau217) and neurobiological risk (NFL, GFAP) were included (hereafter, collectively referred to as biomarkers). These biomarkers were measured using single-molecule array assays. Details on biomarker measurements are provided in eMethods 3 in Supplement 1, and the measurement protocol is described elsewhere.^8^

#### Dementia Assessment

Incidence of dementia was the main outcome variable, with dementia due to AD as secondary. Participants were examined at every data collection wave using structured interviews, clinical examinations, and cognitive testing. Dementia diagnoses were made using *Diagnostic and Statistical Manual of Mental Disorders* (Fourth Edition) criteria^26^ and AD dementia diagnosis according to the National Institute of Neurological and Communicative Disorders and Stroke–Alzheimer’s Disease and Related Disorders Association criteria,^27^ following a validated 3-step procedure: preliminary diagnosis by 2 independent physicians and a final opinion from a senior neurologist (G.G. or L.F.) in case of disagreement. For individuals who died between follow-up examinations, clinical records and death certificates were obtained and reviewed by the same physicians involved in the initial diagnostic process to determine whether participants died with dementia.

#### Other Variables

We considered several factors that may confound the association between diet quality and dementia risk, including (1) sociodemographic variables (sex, age, longest occupation held in life, living arrangement, and highest educational level), (2) lifestyle variables (smoking status, light and moderate physical activity levels, body mass index [calculated as weight in kilograms divided by height in meters squared], and energy intake [in kilocalories per day]), and (3) morbidities (diabetes, heart disease, cerebrovascular disease, depression and other mood disorders, hypertension, anemia, and chronic kidney disease).^28^

To assess potential effect modifiers of the study associations, we used data on sex; age; and apolipoprotein E (*APOE*), which was extracted from DNA using peripheral blood samples and genotyped in a subset of participants. Participants with at least 1 ε4 allele were considered *APOE*-ε4 carriers. In ancillary analyses, we used cognitive impairment, no dementia as an exclusion criterion, defined as scoring 1.5 SDs below age-specific means on 1 or more cognitive domains in the absence of a dementia diagnosis.^29^ Data on race and ethnicity were unavailable and therefore not included in the analyses.

### Statistical Analysis

Data were analyzed between September 2024 and August 2025 and reanalyzed in March and April 2026. Baseline characteristics were summarized by dichotomized biomarker categories (higher and lower), using previously established cutoffs predictive of clinical dementia.^8^ Differences were examined using analysis of variance for continuous variables and χ^2^ tests for categorical variables.

Participants were followed up from baseline until incident dementia, death, loss to follow-up, or the end of follow-up. We used Cox proportional hazards regression to estimate hazard ratios (HRs) and 95% CIs for incident dementia. To study the long-term association of diet and to reduce the measurement error of diet and covariates, we used the cumulative average adherence to the dietary patterns and cumulative average levels of continuous potential confounders and the most recent information on categorical potential confounders. Details of the main analysis and cumulative estimations are provided in eMethods 4 in Supplement 1.

The cumulative average adherence to each dietary pattern was standardized (*z* scores) and modeled as (1) a continuous variable and (2) restricted cubic splines (using the median as reference). The levels of each biomarker were dichotomized, as previously described.^8^ *P* values for 2-way multiplicative interactions between adherence to dietary patterns and biomarker levels were calculated using Wald tests. We report the results from Cox regression models adjusted for prespecified confounders. Missing covariate data were handled by using multiple imputations with chained equations and averaging the results from 10 imputed datasets. The proportional hazards assumption was assessed using both global and covariate-specific Schoenfeld residual–based tests. Given that violations were detected for light physical activity, stratified Cox models were fitted (ie, assuming equal HRs across strata but a baseline hazard unique to each strata).

The 10-year cumulative incidence of dementia, accounting for the competing risk of death, was examined using the cumulative incidence function (CIF). To test the robustness of our results, we conducted several sensitivity analyses, as well as subgroup analyses. The ancillary analyses are described in eMethods 5 in Supplement 1.

All tests were 2-sided, with *P* < .05 considered statistically significant. Statistical significance was primarily defined at the nominal level (*P* < .05) and alternatively at a Bonferroni-adjusted threshold to account for multiple comparisons in main (*P* < .05/9) and subgroup (*P* < .05/27) analyses. The descriptive and main analyses were conducted using Stata, version 17.0 (StataCorp LLC). The CIF and restricted mean time lost (RMTL) analyses were performed using R, version 4.5.0 packages khsmisc, ipw, and survival (R Foundation for Statistical Computing).

## Results

### Descriptive Results

From the initial SNAC-K sample of 3363 participants, we excluded institutionalized participants (n = 191); those with dementia (n = 105) or Parkinson disease (n = 31); and those with missing data on dementia (n = 10), serum biomarkers (n = 778), or baseline dietary information (n = 383). Our final study sample consisted of 1865 participants (mean [SD] age at baseline, 70.5 [9.3] years; 1125 female [60.3%] and 740 male [39.7%]). A flow diagram on losses to follow-up is presented in eFigure 1 in Supplement 1, and a comparison of participants included in the analysis with those excluded is provided in eTable 1 in Supplement 1. A total of 720 participants (38.6%) had a university education, 356 (19.1%) were lifelong manual workers, and 915 (49.1%) lived alone. Baseline characteristics of the analytic sample are reported in Table 1.

**Table 1.** Baseline Characteristics of the Study Participants by Levels of Blood-Based Biomarkers of AD (p-tau217) and Nonspecific Neurodegeneration (NFL, GFAP) (N = 1865)

Characteristic	Participants, No. (%)					
	p-tau217^a^		NFL^a^		GFAP^a^	
	High (n = 589)	Low (n = 1276)	High (n = 668)	Low (n = 1194)	High (n = 647)	Low (n = 1218)
**Dietary pattern adherence**						
AMED, median (IQR)	−0.18 (−0.76 to 0.39)	0.20 (−0.74 to 0.97)	−0.16 (−0.74 to 0.42)	0.39 (−0.76 to 0.97)	−0.16 (−0.72 to 0.44)	0.40 (−0.76 to 0.98)
AHEI, median (IQR)	−0.14 (−0.91 to 0.56)	0.12 (−0.54 to 0.73)	−0.22 (−0.92 to 0.48)	0.16 (−0.53 to 0.79)	−0.17 (−0.85 to 0.48)	0.15 (−0.56 to 0.79)
rEDII, median (IQR)	−0.08 (−0.72 to −0.44)	0.12 (−0.38 to 0.64)	−0.09 (−0.67 to −0.41)	0.14 (−0.38 to 0.66)	−0.06 (−0.63 to 0.44)	0.13 (−0.41 to 0.67)
**Sociodemographic**						
Sex						
Female	330 (56.0)	795 (62.3)	412 (61.6)	713 (59.6)	445 (68.8)	680 (55.8)
Male	259 (44.0)	481 (37.0)	257 (38.4)	483 (40.4)	202 (31.2)	538 (44.2)
Age, median (IQR), y	78.2 (72.0 to 81.8)	66.2 (60.5 to 72.3)	78.3 (72.3 to 84.2)	66.2 (60.4 to 72.2)	78.2 (72.1 to 81.8)	66.2 (60.4 to 72.2)
Manual worker	133 (22.6)	223 (17.5)	156 (23.3)	200 (16.7)	151 (23.3)	205 (16.8)
Living arrangement, alone	328 (55.7)	587 (46.1)	396 (59.3)	519 (43.5)	376 (58.1)	539 (44.4)
University education	193 (32.8)	527 (41.3)	188 (28.1)	532 (44.5)	355 (54.9)	554 (45.5)
**Lifestyle**						
Current tobacco smoking	220 (17.2)	73 (12.4)	209 (17.5)	84 (12.6)	220 (18.1)	73 (11.3)
Daily light physical activity	50 (8.5)	144 (11.3)	51 (7.6)	143 (12.0)	52 (8.0)	142 (11.7)
Daily moderate to vigorous physical activity	59 (10.0)	229 (18.0)	64 (9.6)	224 (18.7)	74 (11.4)	214 (17.6)
BMI, median (IQR)	25.1 (23.0 to 27.7)	25.8 (23.5 to 28.4)	24.8 (22.6 to 27.5)	25.9 (23.8 to 28.7)	24.8 (22.6 to 27.7)	25.8 (23.7 to 28.4)
Energy intake, median (IQR), kcal/d	2011 (1546 to 2538)	1868 (1496 to 2327)	1942 (1541 to 2438)	1890 (1500 to 2363)	1911 (1495 to 2422)	1904 (1509 to 2374)
**Morbidities**						
Diabetes	67 (11.4)	85 (6.7)	74 (11.1)	78 (6.5)	62 (9.6)	90 (7.4)
Heart disease	193 (32.8)	170 (13.3)	214 (32.0)	149 (12.5)	171 (26.4)	192 (15.8)
Cerebrovascular disease	40 (6.8)	44 (3.5)	48 (7.2)	36 (3.0)	43 (6.7)	41 (3.4)
Depression	39 (6.6)	103 (8.1)	49 (7.3)	93 (7.8)	45 (7.0)	97 (8.0)
Hypertension	422 (72.7)	855 (67.0)	494 (73.8)	783 (65.5)	481 (74.3)	796 (65.4)
Anemia	88 (14.9)	61 (4.8)	109 (16.3)	40 (3.3)	82 (12.7)	67 (5.5)
Chronic kidney disease	371 (63.0)	334 (26.2)	471 (70.4)	234 (19.6)	413 (63.8)	292 (24.0)
**Cognitive**						
CIND^b^	119 (22.5)	222 (18.7)	130 (22.0)	211 (18.8)	134 (23.2)	207 (18.2)
*APOE*-ε4^b^	200 (34.0)	335 (26.3)	171 (25.6)	364 (30.4)	182 (28.1)	353 (29.0)
All-cause dementia	140 (23.8)	100 (7.8)	155 (23.2)	85 (7.1)	151 (23.3)	89 (7.3)
AD dementia	72 (12.3)	53 (4.15)	84 (12.6)	41 (3.4)	82 (12.8)	43 (3.5)

Abbreviations: AD, Alzheimer disease; AHEI, Alternative Healthy Eating Index; AMED, Alternate Mediterranean Diet Index; *APOE*, apolipoprotein E; BMI, body mass index (calculated as weight in kilograms divided by height in meters squared); CIND, cognitive impairment, no dementia; GFAP, glial fibrillary acidic protein; NFL, neurofilament light chain; p-tau217, phosphorylated tau at threonine 217; rEDII, reversed Empirical Dietary Inflammatory Index.

^a^ Biomarker levels: p-tau217 high, 0.13 to 6.92 pg/mL; p-tau217 low, 0 to 0.13 pg/mL; NFL high, from 20.18 to 389.05 pg/mL; NFL low, 3.06 to 20.17 pg/mL; GFAP high, 142.58 to 6143.93 pg/mL; GFAP low, 14.17 to 142.14 pg/mL.

^b^ Missing data: *APOE*-ε4, 44 participants; CIND, 207 participants.

Across the dietary patterns, the rEDII showed a low correlation with the AHEI (*r* = 0.32) and a very low correlation with the AMED (*r* = 0.11). The AHEI was moderately correlated with the AMED (*r* = 0.60). There was also some degree of overlap across biomarkers, with 827 participants (44.3%) having low levels of p-tau217, NFL, and GFAP, while 262 (14.0%) had high levels of all 3 (eFigure 2 in Supplement 1).

### Main Results

**Figure 1.**
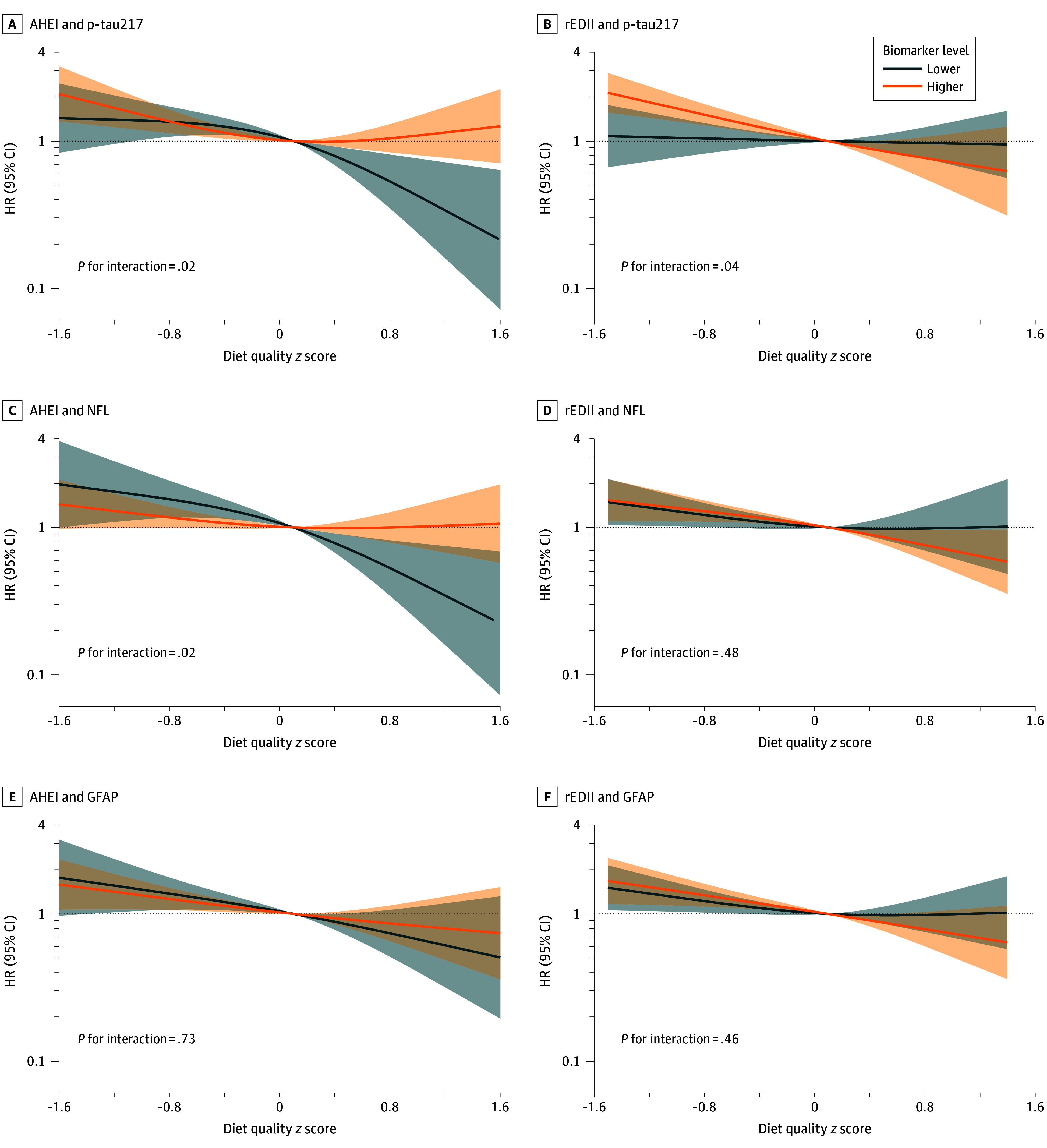
Line Graphs of Spline Associations of Cumulative Diet Quality With 15-Year Dementia Risk, Stratified by Blood-Based Biomarkers of Alzheimer Disease Pathology (Phosphorylated Tau at Threonine 217 [p-tau217]) and Nonspecific Neurodegeneration (Neurofilament Light Chain [NFL], Glial Fibrillary Acidic Protein [GFAP]) (N = 1865) Biomarker levels: p-tau217 high, 0.13 to 6.92 pg/mL; p-tau217 low, 0 to 0.13 pg/mL; NFL high, from 20.18 to 389.05 pg/mL; NFL low, 3.06 to 20.17 pg/mL; GFAP high, 142.58 to 6143.93 pg/mL; GFAP low, 14.17 to 142.14 pg/mL. Results were obtained from Cox proportional hazards regression models. Diet patterns were standardized and modeled as 3-knot restricted cubic splines. Hazard ratios (HRs) and 95% CIs were plotted for adherence to the dietary patterns above the 1st percentile and below the 99th percentile and obtained from models with interaction terms between the dietary pattern and biomarker level. Models were adjusted for sociodemographic variables, lifestyle variables, and morbidities as outlined in the Methods. AHEI indicates Alternative Healthy Eating Index; rEDII, reversed Empirical Dietary Inflammatory Index.

During the 15-year follow-up (mean [SD], 8.4 [4.0] years; range, <0.1 to 15.9 years), 240 participants developed dementia (incidence rate, 15.4 per 1000 person-years; 95% CI, 13.5-17.4 per 1000 person-years). In participants with higher biomarker levels, even when suggesting possible AD pathology, adherence to healthier dietary patterns was associated with a lower risk of dementia, although several comparisons were not significant (Table 2). A 1–*z*-score increase in the rEDII was associated with lower dementia risk among participants with elevated p-tau217, NFL, and GFAP levels, with HRs of 0.71 (95% CI, 0.58-0.88), 0.79 (95% CI, 0.66-0.95), and 0.73 (95% CI, 0.60-0.89), respectively. The opposite pattern was observed for the AMED and AHEI. Comparisons between dementia and high biomarker levels were generally nonsignificant, while associations were found among participants with lower biomarker levels. Differences between biomarker levels were especially marked for AHEI and p-tau217 (*P* for interaction = .04) and NFL (*P* for interaction = .008). Spline analyses were broadly consistent with these findings (Figure 1; eFigure 3 in Supplement 1) and only suggested nonlinearity of the associations between AHEI and dementia risk among participants with lower p-tau217 (*P* for nonlinearity = .046) and NFL (*P* for nonlinearity = .050) levels. Analyses using AD-related dementia (125 participants [6.7%]) and competing-risk models yielded similar results to those observed for all-cause dementia (eTables 5 and 6 in Supplement 1).

**Table 2.** Linear Association of Cumulative Diet Quality With 15-Year Dementia Risk, Stratified by Blood-Based Biomarkers of AD (p-tau217) and Nonspecific Neurodegeneration (NFL, GFAP) (N = 1865)^a^

Biomarker level^b^	Dietary pattern					
	AMED		AHEI		rEDII	
	HR (95% CI)	*P* value for interaction	HR (95% CI)	*P* value for interaction	HR (95% CI)	*P* value for interaction
**p-tau217**						
Higher (n = 589)	0.89 (0.71-1.11)	.35	0.80 (0.69-1.00)^c^	.04	0.71 (0.58-0.88)^d^	.007
Lower (n = 1276)	0.77 (0.60-0.96)^c^		0.66 (0.58-0.88)^d^		0.97 (0.80-1.20)	
**NFL**						
Higher (n = 669)	0.92 (0.75-1.14)	.10	0.89 (0.72-1.04)	.008	0.79 (0.66-0.95)^c^	.48
Lower (n = 1196)	0.71 (0.54-0.92)^c^		0.57 (0.44-0.73)^d^		0.88 (0.70-1.10)	
**GFAP**						
Higher (n = 647)	0.90 (0.73-1.11)	.25	0.74 (0.69-1.02)	.71	0.73 (0.60-0.89)^c^	.13
Lower (n = 1218)	0.74 (0.58-0.97)^c^		0.79 (0.63-0.99)^c^		0.91 (0.74-1.11)	

Abbreviations: AD, Alzheimer disease; AHEI, Alternative Healthy Eating Index; AMED, Alternate Mediterranean Diet Index; GFAP, glial fibrillary acidic protein; HR, hazard ratio; NFL, neurofilament light chain; p-tau217, phosphorylated tau at threonine 217; rEDII, reversed Empirical Dietary Inflammatory Index.

^a^ Results were obtained from Cox proportional hazards regression models. Diet indices were standardized and modeled linearly. Hazard ratios and 95% CIs were obtained from models with interaction terms between the dietary pattern and biomarker level. Models were adjusted for sociodemographic variables, lifestyle variables, and morbidities, as outlined in the Methods.

^b^ Biomarker levels: p-tau217 high, 0.13 to 6.92 pg/mL; p-tau217 low, 0 to 0.13 pg/mL; NFL high, from 20.18 to 389.05 pg/mL; NFL low, 3.06 to 20.17 pg/mL; GFAP high, 142.58 to 6143.93 pg/mL; GFAP low, 14.17 to 142.14 pg/mL.

^c^ *P* < .05 (nominally significant).

^d^ *P* < .05/9 (Bonferroni corrected).

### Ancillary and Sensitivity Analyses

**Figure 2.**
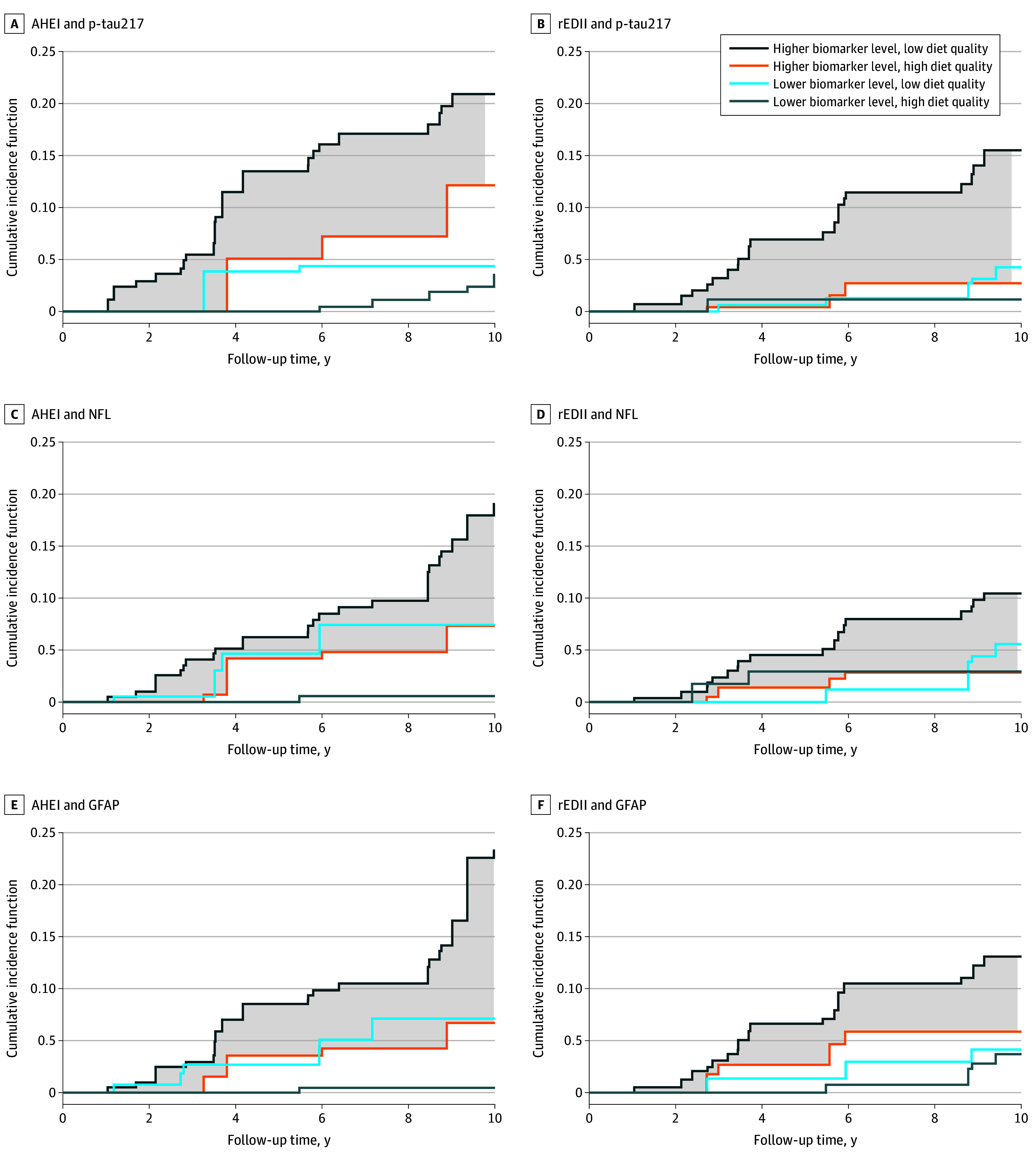
Line Graphs of Cumulative Incidence of Dementia, Stratified by Cumulative Diet Quality and Levels of Blood-Based Biomarkers of Alzheimer Disease (Phosphorylated Tau at Threonine 217 [p-tau217]) and Nonspecific Neurodegeneration (Neurofilament Light Chain [NFL], Glial Fibrillary Acidic Protein [GFAP]) (N = 1865) Biomarker levels: p-tau217 high, 0.13 to 6.92 pg/mL; p-tau217 low, 0 to 0.13 pg/mL; NFL high, from 20.18 to 389.05 pg/mL; NFL low, 3.06 to 20.17 pg/mL; GFAP high, 142.58 to 6143.93 pg/mL; GFAP low, 14.17 to 142.14 pg/mL. Results were obtained from the Aalen-Johansen estimator, accounting for the competing risk of death and adjusted for sociodemographic variables, lifestyle variables, and morbidities, as outlined in the Methods. Shading indicates 10-year restricted mean time lost difference between lower and higher diet quality among the participants with higher levels of the biomarkers. AHEI indicates Alternative Healthy Eating Index; rEDII, reversed Empirical Dietary Inflammatory Index.

The analyses of RMTL due to dementia over a 10-year period revealed less dementia-free time lost among the participants with higher biomarker levels who adhered to healthier diets (Figure 2; eFigure 4 in Supplement 1). In participants with higher baseline levels of p-tau217, NFL, and GFAP, higher rEDII scores were associated with shorter RMTL, with differences of −0.89 years (95% CI, −1.50 to −0.29 years), −0.34 years (95% CI, −0.90 to −0.18 years), and −0.53 years (95% CI, −0.91 to −0.15 years), respectively (eTable 7 in Supplement 1). Among participants with higher baseline p-tau217 levels, higher adherence to the AMED and AHEI was associated with a similar pattern, with RMTL differences of −0.78 years (95% CI, −1.16 to −0.40 years) and −0.66 years (95% CI, −1.03 to −0.28 years), respectively. Consistently, higher rEDII scores were also associated with a significantly lower 10-year cumulative incidence of dementia among participants with higher baseline levels of p-tau217, NFL, and GFAP, with CIF differences of −0.24 (95% CI, −0.39 to −0.09), −0.16 (95% CI, −0.30 to −0.01), and −0.17 (95% CI, −0.31 to −0.02), respectively (eTable 8 in Supplement 1).

Repeating the main analyses after excluding the 19 participants with likely misreported dietary data (eTable 9 in Supplement 1); excluding the 125 participants with cognitive impairment, no dementia (eTable 10 in Supplement 1); excluding the 82 participants who developed dementia within the first 6.7 years of follow-up (eTable 11 in Supplement 1); using alternative cutoffs for AD biomarker stratification (eTable 12 in Supplement 1); and using the reversed Dietary Inflammatory Index as an alternative operationalization of dietary inflammation (eTable 13 in Supplement 1) rendered comparable results.

### Subgroup Analyses

Some of the joint associations between the blood-based biomarkers and dietary patterns with regard to dementia risk were significantly different across age subgroups (eTable 14 in Supplement 1), *APOE*-ε4 genotypes (1821 participants) (eTable 15 in Supplement 1), and sexes (eTable 16 in Supplement 1). Specifically, an inverse association of AHEI with dementia among participants with higher p-tau217 levels was observed only in those aged 78 years or older (HR, 0.72; 95% CI, 0.56-0.92), an association of rEDII with lower dementia incidence among participants with lower GFAP levels emerged in *APOE*-ε4 noncarriers (HR, 0.70; 95% CI, 0.57-0.86), and the inverse association of AMED with dementia was observed in men with higher NFL levels (HR, 0.61; 95% CI, 0.42-0.88).

## Discussion

This 15-year cohort study of older adults without dementia at baseline found that higher diet quality was associated with lower dementia risk and with a delayed onset of dementia, even among participants with elevated serum levels of p-tau217, NFL, and GFAP, a group at increased risk for dementia.^8^ In this subset of participants at risk, the rEDII showed associations across all 3 dietary patterns. Our findings were robust in several sensitivity analyses.

To our knowledge, this study is the first to show that diet quality may modify the relationship between AD pathology or broader neurobiological and dementia risks. Previous longitudinal studies have consistently shown healthier diets to be associated with brain health.^3,15,29^ Higher adherence to the Mediterranean diet has been specifically associated with a reduction in (1) the accumulation of AD biomarkers, such as β-amyloid and tau tangles,^30^ and (2) global AD pathology,^31^ including less brain atrophy in areas commonly affected by AD (eg, cingulate cortex, peripheral lobe, temporal lobe, hippocampus)^32^ and later dementia onset.^31^ In contrast, evidence on the AHEI is limited and inconsistent,^33,34^ and studies on the EDII remain scarce.^16,17,35^ Our findings extend this literature by suggesting that healthy dietary patterns may delay dementia onset, including AD-related dementia, and prolong a dementia-free life even in individuals with possible underlying AD pathology. This distinction is notable, as it is well known that the presence of AD pathology increases dementia risk but does not inevitably lead to its clinical manifestation.^4^

An important finding of our study was that the associations between diet quality and onset of dementia varied across dietary patterns (ie, AMED, AHEI, and rEDII). While each pattern highlights specific dimensions of diet quality (note the very low to moderate correlations across patterns), they all share similarities, such as promoting the consumption of vegetables, fruits, nuts, and whole grains and discouraging consumption of red and processed meats and sugar-sweetened beverages. In a previous cross-sectional study from the SNAC-K cohort, the Mediterranean diet showed associations with AD pathology biomarkers (eg, p-tau181) and broader neurobiological risk biomarkers (eg, NFL, GFAP) at lower, intermediate, and higher levels of their distribution, while the EDII only did so at higher biomarker levels.^36^ These findings were partially mirrored in our study, as the associations of the AMED and AHEI with incidence of dementia were observed primarily in participants with lower biomarker levels, while those of the rEDII were more evident among those with higher biomarker levels.

Another study conducted in SNAC-K showed that elevated levels of p-tau, NFL, and GFAP are associated with all-cause and AD dementia incidence out of 6 blood-based biomarkers.^8^ Since p-tau proteins are recognized as key biomarkers of AD pathology,^34^ the different associations of dietary patterns with dementia between p-tau217 levels in our study suggest a specific mechanistic interplay linking diet to amyloid-related processes, which could contribute to dementia risk, particularly AD dementia. With regard to NFL and GFAP, both are non–AD-specific biomarkers of neurodegeneration and neuroinflammation, with NFL indicating general neuronal damage and GFAP suggesting astrocyte activation.^5^ The NFL-specific and GFAP-specific associations of the AHEI with dementia suggest a particular neuroprotective role of this dietary pattern among individuals with lower biomarker levels. These associations may overlap with the pathophysiology between chronic cardiovascular diseases and neurodegenerative disorders, as the AHEI was designed based on cardiovascular dietary risks.^12,13,37^

Several pathways may underlie the association between diet and dementia. First, a number of components of a healthier diet (eg, B vitamins, polyphenols, polyunsaturated fatty acids) may enhance cognitive health and resilience^7,25^ and lead to improved late-life cognitive trajectories.^23,38,39^ Second, inflammatory pathways have been suggested as particularly relevant to neurodegeneration and AD pathology.^35^ In our study, lower dietary inflammatory potential, measured by the rEDII, was consistently associated with lower dementia risk and later onset among participants with higher biomarker levels, in line with cohort studies finding associations between proinflammatory diets and poorer cognitive outcomes, reduced brain volume, and greater dementia risk.^14,19,40,41^ However, the evidence from randomized clinical trials on cognitive function remains inconclusive.^42^ The rEDII captures the food consumption directly correlated with inflammatory biomarkers, such as interleukin 6 and tumor necrosis factor α,^14,17,40^ which have been associated with neurodegeneration^43^ and AD pathology.^44^ Chronic systemic inflammation may promote neuroinflammation, a key driver of neurodegeneration.^4,35,45^ Besides the rEDII, the Mediterranean diet^16,35^ and AHEI^14^ have also been inversely associated with inflammation, possibly indicating a shared pathway to delay or prevent dementia. That the rEDII showed pronounced associations with dementia among the participants with higher p-tau217, NFL, and GFAP levels is indeed in line with the evidence suggesting inflammation as a key pathway.^46^ Finally, it should be noted that other mechanisms, such as oxidative stress, insulin resistance, lipid metabolism dysregulation, and the gut-brain axis, may also link diet with neurodegeneration and dementia.^47^

In our study, we observed some differences in dementia risk in the joint association between AD biomarkers and diet quality across age subgroups, the *APOE*-ε4 genotypes, and sex, 3 key unmodifiable risk factors for dementia and dementia due to AD. First, in the participants with higher p-tau217 levels, the beneficial associations of the AHEI were only observed in those aged 78 years or older, which may be due to the faster AD pathology progression at earlier ages, making the potential buffer effect of a healthier diet less evident.^48^ Second, the finding in participants with lower GFAP levels of an association between higher diet quality (measured with the rEDII) and dementia onset only among *APOE*-ε4 noncarriers is in line with previous studies and could be explained by the *APOE*-ε4 variant^48,49^ compromising neuroanatomic reserve, increasing the permeability of the blood-brain barrier and proinflammatory cytokine production, and hindering repair mechanisms.^49,50^ Finally, the association between AMED and dementia risk only among men with high NFL concentrations is compatible with sex differences in cardiovascular contributions to dementia risk, which may also shape how diet translates into brain-related benefits.^51,52,53,54,55,56,57,58,59^

### Strengths and Limitations

The relatively large population-based sample of older adults and the 15-year follow-up period are arguably strengths of our study. The SNAC-K also included a rigorous, extensive, in-person evaluation and linkage to patient charts and death registers to assess dementia and other comorbidities. Moreover, our study not only used a panel of AD and neurodegeneration biomarkers, including p-tau217, but also analyzed the associations of 3 different dietary patterns with dementia, which allowed for side-by-side comparisons. Not focusing on single nutrients or food groups (but rather on dietary patterns) allowed us to account for the complex interrelationships among them.

Nevertheless, our study must be considered together with several limitations. First, dietary information and several adjustment variables were self-reported, which may have caused potential misclassification and residual confounding. Second, despite dietary patterns reflecting the joint impact of foods and nutrients, they do not allow for disentangling the role of specific components that underlie the observed associations. Third, dietary information was not available for all participants in the first 2 follow-up waves and for none beyond that point, limiting a fully time-varying assessment of diet. Fourth, in our study, biomarkers were measured in serum, which may have different bioavailability from plasma and cerebrospinal fluid.^6^ Fifth, other blood-based assays may perform better than single-molecule array assays for predicting AD.^32^ Sixth, to categorize biomarker levels, we used cutoffs developed in a previous study of the same cohort.^8^ Seventh, missing dietary and biomarker data were substantial, and excluded participants were generally older, were less educated, and had more comorbidities. Finally, the study sample consisted of community-dwelling, urban, and relatively highly educated and affluent older adults from Stockholm, Sweden. Although data on participants’ race and ethnicity were not collected in SNAC-K, it is likely that the majority were White. The limited heterogeneity across these sociodemographic variables, of which several are established determinants for both dietary intake and dementia, restricts the generalizability of our findings to more diverse populations and settings.^2,55^

## Conclusions

This 15-year cohort study of older adults found that greater adherence to healthier dietary patterns was associated with a reduced risk of dementia, including among participants with AD-related pathology and broader neurobiological risk. In this higher risk group, lower inflammatory potential of diet showed the most consistent inverse associations. Our findings were robust in several sensitivity analyses.

These results reinforce the importance of dietary interventions for dementia, not only for the general population but also for individuals already at elevated disease risk, and support the development of precision public health strategies and personalized dietary recommendations in clinical practice. Future studies should confirm these associations in more diverse populations and identify the specific foods and nutrients driving the observed benefits.

## Notes

Supplement 1. **eMethods 1. **Study Design **eMethods 2. **Dietary Pattern Adherence Estimation **eMethods 3. **Biomarker Measurements **eMethods 4. **Main Analysis Setup and Cumulative Updating of Diet and Covariates **eMethods 5. **Ancillary and Sensitivity Analyses **eTable 1. **Characteristics of Participants in the Study Sample and Excluded Sample **eTable 2. **The Alternate Mediterranean Diet Index (AMED) Scoring Method and Components Consumption Median at Baseline **eTable 3. **The Alternative Healthy Eating Index (AHEI) Scoring Method and Components Consumption Mean at Baseline **eTable 4. **Consumption and Inflammatory Effect Scores of the Components of the Empirical Dietary Inflammatory Index (EDII) **eTable 5. **Sensitivity Analysis of the Association of Blood-Based Biomarkers of Alzheimer Disease With 15-Year Dementia Due to Alzheimer Disease Risk, Stratified by Levels of Cumulative Diet Quality (Adherence to the AMED, AHEI, and EDII) **eTable 6.** Sensitivity Analysis of the Association of Cumulative Diet Quality With 15-Year Dementia Due to Alzheimer Disease Risk, by Levels of Blood-Based Biomarkers of Alzheimer Disease-Related Pathology (p-tau217) and Nonspecific Neurodegeneration (NFL, GFAP) in a Competitive Risk Setting **eTable 7.** Ancillary Analysis Estimating the 10-Year Restricted Mean Time Lost at 10th and 90th Percentile of Average Diet Quality, by Levels of the Biomarkers of Alzheimer Disease-Related Pathology (p-tau217) and Nonspecific Neurodegeneration (NFL, GFAP) **eTable 8.** Ancillary Analysis Estimating the 10-Year Cumulative Incidence Function at 10th and 90th Percentile of Average Diet Quality, by Levels of the Biomarkers of Alzheimer Disease-Related Pathology (p-tau217) and Nonspecific Neurodegeneration (NFL, GFAP) **eTable 9.** Sensitivity Analysis of the Association of Cumulative Diet Quality With 15-Year Dementia Risk, by Blood-Based Biomarkers of Alzheimer Disease-Related Pathology (p-tau217) and Nonspecific Neurodegeneration (NFL, GFAP), Excluding Likely Misreported Dietary Data **eTable 10.** Sensitivity Analysis of the Association of Cumulative Diet Quality With 15-Year Dementia Risk, by Blood-Based Biomarkers of Alzheimer Disease-Related Pathology (p-tau217) and Nonspecific Neurodegeneration (NFL, GFAP), Excluding Participants With Cognitive Impairment, No Dementia **eTable 11. **Sensitivity Analysis of the Association of Cumulative Diet Quality With 15-Year Dementia Risk, by Blood-Based Biomarkers of Alzheimer Disease-Related Pathology (p-tau217) and Nonspecific Neurodegeneration (NFL, GFAP), Excluding Participants Who Developed Dementia During the First and Second Follow-Up **eTable 12.** Sensitivity Analysis of the Association of Cumulative Diet Quality With 15-Year Dementia Risk, by Blood-Based Biomarkers of Alzheimer Disease-Related Pathology (p-tau217) and Nonspecific Neurodegeneration (NFL, GFAP), Using Highest Tertile of Biomarkers as a Cutoff **eTable 13.** Sensitivity Analysis of the Association of Cumulative Adherence to the Reversed Dietary Inflammatory Index With 15-Year Dementia Risk, Stratified by Blood-Based Biomarkers of Alzheimer Disease-Related Pathology (p-tau217) and Nonspecific Neurodegeneration (NFL, GFAP) **eTable 14.** Subgroup Analysis of the Association of Cumulative Diet Quality With 15-Year Dementia Onset, by Levels of Blood-Based Biomarkers of Alzheimer Disease-Related Pathology (p-tau217), Nonspecific Neurodegeneration (NFL, GFAP), and Age ** eTable 15.** Subgroup Analysis of the Association of Cumulative Diet Quality With 15-Year Dementia Onset, by Levels of Blood-Based Biomarkers of Alzheimer Disease-Related Pathology (p-tau217), and Nonspecific Neurodegeneration (NFL, GFAP), and *APOE*-e4 ** eTable 16.** Subgroup Analysis of the Association of Cumulative Diet Quality With 15-Year Dementia Onset, by Levels of Blood-Based Biomarkers of Alzheimer Disease-Related Pathology (p-tau217), Nonspecific Neurodegeneration (NFL, GFAP), and Sex **eFigure 1.** Flow Diagram of Study Sample Selection **eFigure 2.** Overlap of High Levels of Blood-Based Biomarkers of Alzheimer Disease-Related Pathology (p-tau217) and Nonspecific Neurodegeneration (NFL, GFAP) **eFigure 3.** Spline Associations of Cumulative Adherence to Alternate Mediterranean Diet (AMED) With 15-Year Dementia Risk, Stratified by Blood-Based Biomarkers of Alzheimer Disease-Related Pathology (p-tau217) and Nonspecific Neurodegeneration (NFL, GFAP) **eFigure 4.** Cumulative Incidence of Dementia, Stratified by Diet Quality and Levels of Blood-Based Biomarkers of Alzheimer Disease-Related Pathology (p-tau217) and Nonspecific Neurodegeneration (NFL, GFAP) and 10-Year Restricted Mean Time Lost Difference Between Lower and Higher Diet Quality Among the Participants With Higher Levels of the Biomarkers
eReferences.

Supplement 2.
Data Sharing Statement
