# Supplementary material for: Diet Quality and Dementia Risk in Older Adults With Alzheimer Pathology
Source: JAMA Netw Open. 2026 Jun 25;9(6):e2620254. doi: 10.1001/jamanetworkopen.2026.20254 (PMC13306305; doi:10.1001/jamanetworkopen.2026.20254)
Supplement: Supplement 1. — eMethods 1. Study Design eMethods 2. Dietary Pattern Adherence Estimation eMethods 3. Biomarker Measurements eMethods 4. Main Analysis Setup and Cumulative Updating of Diet and Covariates eMethods 5. Ancillary and Sensitivity Analyses eTable 1. Characteristics of Participants in the Study Sample and Excluded Sample eTable 2. The Alternate Mediterranean Diet Index (AMED) Scoring Method and Components Consumption Median at Baseline eTable 3. The Alternative Healthy Eating Index (AHEI) Scoring Method and Components Consumption Mean at Baseline eTable 4. Consumption and Inflammatory Effect Scores of the Components of the Empirical Dietary Inflammatory Index (EDII) eTable 5. Sensitivity Analysis of the Association of Blood-Based Biomarkers of Alzheimer Disease With 15-Year Dementia Due to Alzheimer Disease Risk, Stratified by Levels of Cumulative Diet Quality (Adherence to the AMED, AHEI, and EDII) eTable 6. Sensitivity Analysis of the Association of Cumulative Diet Quality With 15-Year Dementia Due to Alzheimer Disease Risk, by Levels of Blood-Based Biomarkers of Alzheimer Disease-Related Pathology (p-tau217) and Nonspecific Neurodegeneration (NFL, GFAP) in a Competitive Risk Setting eTable 7. Ancillary Analysis Estimating the 10-Year Restricted Mean Time Lost at 10th and 90th Percentile of Average Diet Quality, by Levels of the Biomarkers of Alzheimer Disease-Related Pathology (p-tau217) and Nonspecific Neurodegeneration (NFL, GFAP) eTable 8. Ancillary Analysis Estimating the 10-Year Cumulative Incidence Function at 10th and 90th Percentile of Average Diet Quality, by Levels of the Biomarkers of Alzheimer Disease-Related Pathology (p-tau217) and Nonspecific Neurodegeneration (NFL, GFAP) eTable 9. Sensitivity Analysis of the Association of Cumulative Diet Quality With 15-Year Dementia Risk, by Blood-Based Biomarkers of Alzheimer Disease-Related Pathology (p-tau217) and Nonspecific Neurodegeneration (NFL, GFAP), Excluding Likely Misreported Dietary Data eTable 10. Sensit [file jamanetwopen-e2620254-s001.pdf]

Supplemental Online Content

Mrhar A, Carballo-Casla A, Grande G, et al. Diet quality and dementia risk in older adults with Alzheimer pathology. *JAMA Netw Open*. 2026;9(6):e2620254.
doi:10.1001/jamanetworkopen.2026.20254

eMethods 1. Study Design

eMethods 2. Dietary Pattern Adherence Estimation

eMethods 3. Biomarker Measurements

eMethods 4. Main Analysis Setup and Cumulative Updating of Diet and Covariates

eMethods 5. Ancillary and Sensitivity Analyses

eTable 1. Characteristics of Participants in the Study Sample and Excluded Sample

eTable 2. The Alternate Mediterranean Diet Index (AMED) Scoring Method and Components Consumption Median at Baseline

eTable 3. The Alternative Healthy Eating Index (AHEI) Scoring Method and Components Consumption Mean at Baseline

eTable 4. Consumption and Inflammatory Effect Scores of the Components of the Empirical Dietary Inflammatory Index (EDII)

eTable 5. Sensitivity Analysis of the Association of Blood-Based Biomarkers of Alzheimer Disease With 15-Year Dementia Due to Alzheimer Disease Risk, Stratified by Levels of Cumulative Diet Quality (Adherence to the AMED, AHEI, and EDII)

eTable 6. Sensitivity Analysis of the Association of Cumulative Diet Quality With 15-Year Dementia Due to Alzheimer Disease Risk, by Levels of Blood-Based Biomarkers of Alzheimer Disease-Related Pathology (p-tau217) and Nonspecific Neurodegeneration (NFL, GFAP) in a Competitive Risk Setting

eTable 7. Ancillary Analysis Estimating the 10-Year Restricted Mean Time Lost at 10th and 90th Percentile of Average Diet Quality, by Levels of the Biomarkers of Alzheimer Disease-Related Pathology (p-tau217) and Nonspecific Neurodegeneration (NFL, GFAP)

eTable 8. Ancillary Analysis Estimating the 10-Year Cumulative Incidence Function at 10th and 90th Percentile of Average Diet Quality, by Levels of the Biomarkers of Alzheimer Disease-Related Pathology (p-tau217) and Nonspecific Neurodegeneration (NFL, GFAP)

eTable 9. Sensitivity Analysis of the Association of Cumulative Diet Quality With 15-Year Dementia Risk, by Blood-Based Biomarkers of Alzheimer Disease-Related Pathology (p-tau217) and Nonspecific Neurodegeneration (NFL, GFAP), Excluding Likely Misreported Dietary Data

eTable 10. Sensitivity Analysis of the Association of Cumulative Diet Quality With 15-Year Dementia Risk, by Blood-Based Biomarkers of Alzheimer Disease-Related Pathology (p-tau217) and Nonspecific Neurodegeneration (NFL, GFAP), Excluding Participants With Cognitive Impairment, No Dementia

eTable 11. Sensitivity Analysis of the Association of Cumulative Diet Quality With 15-Year Dementia Risk, by Blood-Based Biomarkers of Alzheimer Disease-Related Pathology (p-tau217) and Nonspecific Neurodegeneration (NFL, GFAP), Excluding Participants Who Developed Dementia During the First and Second Follow-Up

© 2026 Mrhar A et al. *JAMA Network Open*.

eTable 12. Sensitivity Analysis of the Association of Cumulative Diet Quality With 15-Year Dementia Risk, by Blood-Based Biomarkers of Alzheimer Disease-Related Pathology (p-tau217) and Nonspecific Neurodegeneration (NFL, GFAP), Using Highest Tertile of Biomarkers as a Cutoff

eTable 13. Sensitivity Analysis of the Association of Cumulative Adherence to the Reversed Dietary Inflammatory Index With 15-Year Dementia Risk, Stratified by Blood-Based Biomarkers of Alzheimer Disease-Related Pathology (p-tau217) and Nonspecific Neurodegeneration (NFL, GFAP)

eTable 14. Subgroup Analysis of the Association of Cumulative Diet Quality With 15-Year Dementia Onset, by Levels of Blood-Based Biomarkers of Alzheimer Disease-Related Pathology (p-tau217), Nonspecific Neurodegeneration (NFL, GFAP), and Age

eTable 15. Subgroup Analysis of the Association of Cumulative Diet Quality With 15-Year Dementia Onset, by Levels of Blood-Based Biomarkers of Alzheimer Disease-Related Pathology (p-tau217), and Nonspecific Neurodegeneration (NFL, GFAP), and *APOE*-e4

eTable 16. Subgroup Analysis of the Association of Cumulative Diet Quality With 15-Year Dementia Onset, by Levels of Blood-Based Biomarkers of Alzheimer Disease-Related Pathology (p-tau217), Nonspecific Neurodegeneration (NFL, GFAP), and Sex

eFigure 1. Flow Diagram of Study Sample Selection

eFigure 2. Overlap of High Levels of Blood-Based Biomarkers of Alzheimer Disease-Related Pathology (p-tau217) and Nonspecific Neurodegeneration (NFL, GFAP)

eFigure 3. Spline Associations of Cumulative Adherence to Alternate Mediterranean Diet (AMED) With 15-Year Dementia Risk, Stratified by Blood-Based Biomarkers of Alzheimer Disease-Related Pathology (p-tau217) and Nonspecific Neurodegeneration (NFL, GFAP)

eFigure 4. Cumulative Incidence of Dementia, Stratified by Diet Quality and Levels of Blood-Based Biomarkers of Alzheimer Disease-Related Pathology (p-tau217) and Nonspecific Neurodegeneration (NFL, GFAP) and 10-Year Restricted Mean Time Lost Difference Between Lower and Higher Diet Quality Among the Participants With Higher Levels of the Biomarkers

eReferences

This supplemental material has been provided by the authors to give readers additional information about their work.

© 2026 Mrhar A et al. *JAMA Network Open*.

eMethods 1. Study Design

The aim of the Swedish National study on Aging and Care in Kungsholmen (SNAC-K) study is to better understand the process of aging and to identify possible preventive strategies to improve health and care in older adults. After the baseline examination, participants have been followed up regularly every 3 years (those aged ≥78 years) or 6 years (those aged <78 years). Our set of analyses includes baseline data on blood-based biomarkers of AD (p-tau217) and neurobiological vulnerability (NFL, GFAP), data on diet and potential confounders from the baseline, 3-year, and 6-year waves, and dementia diagnoses from the baseline, 3-year, 6-year, 9-year, 12-year, and 15-year waves (as well as from medical and death records).

© 2026 Mrhar A et al. *JAMA Network Open*.

eMethods 2. Dietary Pattern Adherence Estimation

Data from a validated 98-item food frequency questionnaire (FFQ) was used to evaluate habitual diet in the previous year. Participants indicated how frequently they consumed each food and beverage by using a nine-point scale (ranging from 'never' to 'four or more times a day') at baseline and a five-point scale (ranging from 'never or a few times per year' to 'two or more times a day') in the 3-year and 6-year follow-up waves. Color photographs to estimate portion sizes, and food composition tables from the Swedish National Food Agency (version 2024-05-29) to estimate energy and nutrient intakes, were used.

Alternative Mediterranean Diet

To assess the adherence to the Mediterranean diet in a non-Mediterranean population, the nine-item AMED scale by Fung et al. (1) was used. Higher consumption (according to the study-specific medians) of vegetables (excluding potatoes), legumes, fruits, nuts, whole grains, fish, and the monounsaturated/saturated fat ratio; lower consumption of red and processed meat; and moderate ethanol intake (5–15 grams/day in females and 15–25 grams/day in males), were scored 1 point, while other consumption received 0 points (1). The possible score range was from 0 to 9, with higher score representing a healthier, Mediterranean-style diet. Detailed information on the components and scoring of the AMED is shown in **eTable 3**.

Alternative Healthy Eating Index

Adherence to the AHEI, a pattern developed to measure diet quality according to the risk of diet-related chronic diseases, was calculated following the procedure by Chiuve et al. (2). The AHEI was the sum of 11 dietary components: six for which higher consumption is considered healthier (vegetables, fruit, whole grains, nuts and legumes, long chain omega-3 fatty acids, and polyunsaturated fatty acids), one component for which moderate intake is deemed better (alcohol), and four presumably unhealthier components (sugar sweetened drinks and fruit juice, red and processed meat, trans fats, and sodium). Each component was scored on a 0 to 10-point scale. The 11 scores were summed to obtain the AHEI, with a theoretical range from 0 to 110. Higher scores represent healthier diets (3,4). Detailed information on the components and scoring of the AHEI is shown in **eTable 4.**

Empirical dietary inflammatory index

We used a scoring method proposed by Tabung et al. (5) to estimate adherence to the EDII, linking diet quality to the inflammatory potential of foods and beverages This dietary pattern consists of 18 components, which were originally associated with higher or lower levels of three inflammatory biomarkers. Processed meat, read meat, organ meat, fish (other than dark-meat fish), vegetables

© 2026 Mrhar A et al. *JAMA Network Open*.

(other than dark yellow vegetables, leafy green vegetables, and tomatoes), refined grains, high-energy beverages, low-energy beverages, and tomatoes were considered pro-inflammatory; while dark yellow vegetables, leafy green vegetables, snacks, fruit juice, pizza, tea, coffee, beer, and wine were deemed anti-inflammatory. The daily consumption of each food group by every participant was multiplied by an inflammatory effect score, ranging from -1175 (maximally anti-inflammatory) to 252 (maximally pro-inflammatory). Finally, the weighted consumption was summed, rescaled by dividing by 1000, and reverse-transformed for interpretability (rEDII), so that higher values represent lower dietary inflammatory potential (i.e. healthier diet quality). Detailed information on the EDII components and its calculation is shown in **eTable 4**.

© 2026 Mrhar A et al. *JAMA Network Open*.

eMethods 3. Biomarker Measurements

At the baseline wave of SNAC-K, peripheral venous blood samples were collected (note that fasting was not mandatory) and centrifugated. For the purpose of subsequent analysis, serum aliquots were stored at -80°C in cryogenic storage vials. AD blood biomarker quantification was performed using single Molecule Array technology (Simoa, Quanterix). Serum p-tau217 was assessed using Simoa ALZpath p-tau217 Advantage PLUS (Quanterix, category number 104570), and NFL and GFAP were assessed using the Simoa Neuro 2-plex B Kit. For each kit, 25 µL of the sample was diluted 1:4. The assays were conducted following the manufacturer's instructions. The Quanterix instrument provided average enzyme per bead values for calibrators, controls, and samples. Curve-fitting, extrapolation of concentrations, and graphical representation were automatically performed with the Quanterix SR-X software, using the calibrators and a four-parameter logistic curve fit.

© 2026 Mrhar A et al. *JAMA Network Open*.

eMethods 4. Main Analysis Setup and Cumulative Updating of Diet and Covariates

Participants involved in the study sample were free from dementia at baseline (2001-2004) and followed up until the diagnosis of dementia, death, loss to follow-up, or the 15-year data collection wave. Participants who received a diagnosis of dementia during the follow-up examinations or died with dementia before the end of the 15-year data collection wave (2016–2019) were computed as incident dementia cases. Participants who did not develop dementia exited the risk pool at the date of last follow-up (except for those who were never followed, who were censored at the date of baseline plus one day).

We used Cox regression models to estimate the hazard ratio (HR) and 95% confidence interval (CI) of dementia incidence. To study the long-term impact of diet on this outcome, and to reduce the measurement error of diet and covariates, we used the cumulative average adherence to the dietary patterns and cumulative average levels of continuous potential confounders (baseline, 3-year, and 6-year waves), and the most recent information on categorical potential confounders (baseline, 3-year, and 6-year waves). For example, for a participant with three dietary assessments available, diet quality at baseline was used for the first observation, the average diet quality between baseline and the 3-year follow-up was used for the second observation, and the average across baseline, the 3-year follow-up, and the 6-year follow-up for the third observation (spanning until the exit date). Note that the values of dietary and confounder variables recorded on or after dementia diagnosis were disregarded, to minimize the risk of reverse causation.

© 2026 Mrhar A et al. *JAMA Network Open*.

eMethods 5. Ancillary Analyses

Restricted Mean Time Lost

The 10-year cumulative incidence of dementia was estimated in the presence of the competing risk of death using the cumulative incidence function (CIF). Restricted Mean Time Lost (RMTL) due to dementia over 10 years was defined as the area under the CIF and represents the average amount of dementia-free time lost within this time horizon. Adjusted absolute differences in CIF and RMTL were estimated by modeling the corresponding 10-year pseudo-values with multivariable linear regression (6,7). Each model included the dichotomized biomarker variable, the standardized adherence to dietary patterns, and their interaction term, with additional adjustment for confounders. From these models, adjusted absolute differences comparing the 90th and 10th percentiles of dietary adherence were estimated within biomarker strata using post-estimation contrasts. Given the challenge of modelling cumulative variables in this set of analyses, we used average values across waves 1 and 3 for dietary patterns and continuous potential confounders, and baseline values for categorical potential confounders.

Sensitivity analyses

Several sensitivity analyses were conducted. First, because cognitive impairment might influence the reporting of food consumption, we excluded the participants with CIND at baseline (and stopped updating dietary data and confounders whenever CIND arose in follow-up assessments). Second, we omitted the first 6.7 years of follow-up (i.e., the whole first and second follow-up waves), not to consider participants with likely prodromal or undiagnosed dementia. Third, to evaluate whether the observed associations reflected overall risk of dementia and/or dementia due to AD, we repeated the primary analyses using AD dementia as the main outcome. Fourth, because non-AD dementia may occur as a competing event for AD dementia, we treated dementia due to other causes as a competing risk and estimated subdistribution hazard ratios for incident AD dementia using Fine–Gray competing-risk models. Fifth, to account for the sample-specific distribution of biomarkers and assess the robustness of our findings across alternative stratification thresholds, we repeated the main analysis using the highest tertiles of biomarker levels as cut-offs. Sixth, to assess robustness to implausible dietary reporting, likely misreported dietary data were disregarded (energy intake >5000 or <800 kcal/day in males and >4000 or <500 kcal/day in females) (8). Finally, to assess the robustness of the findings on the inflammatory potential of diet, we repeated the main analysis using an alternative operationalization of dietary inflammation, specifically the Dietary Inflammatory Index (9) which was reverse-coded to improve interpretability.

Subgroup analyses and interactions

© 2026 Mrhar A et al. *JAMA Network Open*.

We conducted subgroup analyses by stratifying the associations by sex (males and females), age (<78 and ≥78 years), and *APOE-ε4* genotype (non-carriers and carriers). Estimates for each stratum were obtained from Cox models with three-way multiplicative interaction terms between the standardised adherence to the dietary pattern, categories of the biomarker (i.e., higher/lower), and those of the stratification variable. These models included all main effects and lower-order (two-way) interaction terms, in accordance with the effect hierarchy principle. The statistical significance of the three-way interactions was evaluated using Wald tests.

© 2026 Mrhar A et al. *JAMA Network Open*.

eTable 1. Characteristics of Participants in the Study Sample and Excluded Sample

	**Study sample (1865) N (%)**	**Excluded sample (1498) N (%)**
**Dietary**		
AMED [median (IQR)]	-0.15 (-0.72, 0.98)	-015 (-0.72, 0.42)*
AHEI [median (IQR)]	0.08 (0.62, 0.72)	-0.07 (-0.75, 0.61)*
rEDII [median (IQR)]	0.09 (-0.45, 0.62)	-0.04 (-0.60, 0.52)*
**Sociodemographic**		
Sex, male [n (%)]	740 (39.7)	441 (29.4)*
Sex, female [n (%)]	1125 (60.3)	1057 (70.6)*
Age [median (IQR)]	66.7 (60.6, 78.2)	81.2 (72.2, 90.2)*
Manual worker [n (%)]	356 (19.1)	131 (21.1)
Living arrangement, alone [n (%)]	915 (49.1)	1042 (70.3)*
Education level, university [n (%)]	720 (38.6)	215 (34.6)
**Lifestyle**		
Tobacco smoking, current [n (%)]	293 (15.7)	69 (11.1)*
Light physical activity, daily [n (%)]	194 (40.4)	58 (9.3)*
Moderate-to-vigorous physical activity, daily [n (%)]	288 (15.4)	67 (10.8)*
BMI (kg/m2) [median (IQR)]	25.5 (23.3, 28.2)	24.8 (22.2, 27.3)*
Energy intake (kcal/day) [median (IQR)]	1906 (1503, 2385)	1844 (1481, 2342)
**Morbidity**		
Diabetes [n (%)]	152 (8.2)	144 (9.6)
Heart diseases [n (%)]	363 (19.5)	504 (20.8)
Cerebrovascular disease [n (%)]	84 (4.5)	181 (12.1)*
Depression [n (%)]	142 (7.6)	168 (11.2)*
Hypertension [n (%)]	1277 (68.5)	1000 (66.8)
Anemia [n (%)]	79 (4.2)	73 (4.9)
Chronic kidney disease [n (%)]	705 (37.8)	780 (52.1)*
**Cognitive**		
CIND [n (%)]	341 (19.9)	268 (30.5)*
APOE-E4 [n (%)]	535 (82.7)	312 (12.9)*

© 2026 Mrhar A et al. *JAMA Network Open*.

ptau217	0.094 (0.052, 0.16)	0.17 (0.09, 0.31)*
NFL	16.7 (12.1, 24.8)	30.6 (18.8, 50.2)*
GFAP	113.2 (75.2, 168.1)	180.3 (116.3, 274.8)*

** p < 0.05 (nominally significant)*

Missing data: APOE-E4 (n=44 in study sample), CIND (n=207 in study sample, n=1541 in excluded sample), p-tau217 (n= 1926 in excluded sample), NFL (n= 1849 in excluded sample), GFAP (n= 1849 in excluded sample)

BMI: body mass index; IQR: interquartile range; CIND: cognitive impairment, no dementia; APOE: apolipoprotein E.; p-tau217 = phosphorylated tau 217; NFL = neurofilament light; GFAP = glial fibrillary acidic protein; AMED = Alternative Mediterranean Diet Index, AHEI = Alternative Healthy Eating Index, rEDII = reversed Empirical Dietary Inflammatory Index.

© 2026 Mrhar A et al. *JAMA Network Open*.

eTable 2. The Alternate Mediterranean Diet Index (AMED) Scoring Method and Components Consumption Median at Baseline

		Total n=1865
	**Food components**	**Median (IQR)**
1	Vegetables (without potato) (g/day)	193.5 (136.6)
2	Legumes (g/day)	9.9 (8.8)
3	Fruits (g/day)	273.6 (190.2)
4	Nuts (g/day)	0.91 (22.4)
5	Whole-grain products(g/day)	59.4 (37.6)
6	Fish (g/day)	40.9 (27.8)
7	Monounsaturated to saturated fat (ratio)	0.98 (0.21)
8	Red and processed meat (g/day)	63.4 (43.2)
9	Alcohol (g of ethanol/day)	12.9 (13.4)

IQR (interquartile range)

To calculate the AMED score, a value of 0 or 1 was assigned to each of the nine groups, with the study-specific median as the cutoff. For the components (1) vegetables, (2) legumes, (3) fruits, (4) nuts, (5) whole-grain products, (6) fish, and (7) monounsaturated to saturated fat ratio, persons whose consumption was at or above the median were assigned a value of 1. For the component (8) red and processed meat, persons whose consumption was below the median were assigned a value of 1. For alcohol intake, a value of 1 was assigned if consumed between 5 and 15 g of ethanol per day. The total score range was from 0 to 9.

© 2026 Mrhar A et al. *JAMA Network Open*.

eTable 3. The Alternative Healthy Eating Index (AHEI) Scoring Method and Components Consumption Mean at Baseline

	Component	Criteria		Mean (SD)	
		For minimum score (0)	For maximum score (10)	Male n=740	Female n=1125
1	Vegetables, *servings/d*	0	≥5	2.3 (1.8)	3.02 (2.16)
2	Fruit, *servings/d*	0	≥4	1.79 (1.4)	2.29 (1.49)
3	Whole grains, *g/d*	0	90 (males)<br>75 (female)	66.8 (42.4)	54.6 (33.3)
4	Nuts and legumes, *servings/d*	0	≥1	0.1 (0.1)	0.1 (0.09)
5	Long chain omega-3 fatty acids (DHA, EPA), *mg/d*	0	250	378.8 (271.4)	342.6 (257.4)
6	PUFA, *% of energy*	≤2	≥10	5.3 (1.4)	5.3 (1.5)
7	Alcohol, *drinks/d*	≥2.5	0.5-2.0 (male)<br>0.5-1.5 (female)	1.9 (1.6)	0.92 (1.01)
8	Sugar-sweetened beverages and fruit juice, *servings/d*	≥1	0	0.29 (0.5)	0.25 (0.5)
9	Red and processed meat, *servings/d*	≥1.5	0	1.1 (0.8)	0.9 (0.8)
10	Trans fat, *% of energy*	≥4	≤0.5	1.3 (0.5)	1.2 (0.4)
11	Sodium, *mg/d*	Highest decile	Lowest decile	2908.3 (1146.2)	2298.4 (904.4)

Vegetables: one serving is 0.5 cup of vegetables or 1 cup of green leafy vegetables (1 cup = 236.59 g). Fruit: one serving is 1 medium piece of fruit or 0.5 cup of berries (1 cup = 236.59 g). Whole grain: one serving of a 100% whole-grain product (i.e., 0.5 cup of oatmeal or brown rice) contains ;15–20 g of whole grains (per dry weight). Nuts, legumes, and vegetable protein: one serving is 1 oz (1 oz = 28.35 g) of nuts or 1 tablespoon (15 mL) of peanut butter. Polyunsaturated fatty acids (PUFA): the highest score to individuals with 10% of total energy intake from PUFA. PUFA does not include eicosapentaenoic acid (EPA) or docosahexaenoic acid (DHA) intake. Alcohol: nondrinkers received a score of 2.5. Because the health effects of alcohol are seen at lower quantities in women than in men, we used gender-specific cutoffs. One drink is 4 oz of wine, 12 oz of beer, or 1.5 oz of liquor (1 oz = 28.35 g). Sugar-sweetened beverages: one serving is 8 oz (1 oz = 28.35 g). Red meat and processed meat: one serving is 4 oz of unprocessed meat or 1.5 oz of processed meat (1 oz = 28.35 g). Trans-isomeres: Cutoffs are consistent with original AHEI cutoffs for trans-fat. Soudium: Values in lowest decile were 1700 mg/d in women and 1706 mg/d in men and in highest decile were 3372 mg/d in women and 4201 mg/d in men at baseline.

© 2026 Mrhar A et al. *JAMA Network Open*.

eTable 4. Consumption and Inflammatory Effect Scores of the Components of the Empirical Dietary Inflammatory Index (EDII)

		Total N=1865 Mean (SD)	Overall inflammatory effect score
**Pro-inflammatory EDII components (portions/day)** [a]			
1	Processed meat	0.646 (0.697)	165.03
2	Red meat	0.274 (0.216)	140.19
3	Organ meat	0.030 (0.047)	144.61
4	Fish (other than dark-meat fish)	0.207 (0.151)	252.45
5	Vegetables (other than dark yellow and leafy green)	0.803 (0.653)	136.14
6	Refined grains	1.299 (0.955)	81.21
7	High-energy beverages	0.263 (0.511)	156.85
8	Low-energy beverages	0.030 (0.090)	94.77
9	Tomatoes	0.639 (0.619)	167.92
**Anti-inflammatory EDII components (portions/day)** [b]			
10	Beer	0.347 (0.511)	–136.99
11	Wine	0.324 (0.378)	–249.70
12	Tea	0.661 (0.823)	–42.25
13	Coffee	1.694 (1.082)	–83.18
14	Dark yellow vegetables	0.474 (0.514)	–165.37
15	Leafy green vegetables	0.656 (0.652)	–190.29
16	Snacks	0.139 (0.217)	–45.08
17	Fruit juice	0.378 (0.502)	–58.95
18	Pizza	0.022 (0.041)	–1175.21

The mean daily consumption of each food group by each participant was multiplied by the corresponding inflammatory effect score. The weighted consumption was summed to obtain the EDII and divided by 1000.

[a] Wine = red or white wine. Coffee = soluble, espresso, Italian, or filtered coffee. Dark yellow vegetables = carrots, yellow (winter) squash, yams, or sweet potatoes. Leafy green vegetables = cooked or raw spinach, iceberg or head lettuce, or romaine or leaf lettuce. Snacks = potato chips, corn chips, popcorn, or crackers. Fruit juice = apple juice or cider, orange juice, grapefruit juice, or other fruit juice.

© 2026 Mrhar A et al. *JAMA Network Open*.

[b] Processed meat = processed meats, bacon, or hot dog. Red meat = beef, pork, lamb, or hamburger patty. Organ meat = beef, calf, or pork liver, or chicken or turkey liver. Fish (other than dark-meat fish) = canned tuna, shrimp, lobster, scallops, fish, or other seafood other than dark-meat fish. Other vegetables = celery, mushrooms, green pepper, corn, mixed vegetables, eggplant, zucchini, alfalfa sprouts, or cucumber. Refined grains = white bread, English muffin, bagel or roll, muffin or biscuit, pancakes, or waffles; or white rice; or pasta. High-energy beverages = cola with sugar, other carbonated beverages with sugar, or fruit punch drinks. Low-energy beverages = low-energy cola or other low-energy carbonated beverages. Tomatoes = fresh tomato, tomato juice, or tomato sauce

© 2026 Mrhar A et al. *JAMA Network Open*.

eTable 5. Sensitivity Analysis of the Association of Blood-Based Biomarkers of Alzheimer Disease With 15-Year Dementia Due to Alzheimer Disease Risk, Stratified by Levels of Cumulative Diet Quality (Adherence to the AMED, AHEI, and EDII) (n = 1865)

		Dietary pattern, HR (95% CI)					
		AMED		AHEI		rEDII	
			p-int		p-int		p-int
ptau217	Higher (n=589)	0.83 (0.67, 1.02)	0.227	0.85 (0.66, 1.09)	0.201	0.63 (0.51, 0.80)**	0.037
	Lower (n=1276)	0.95 (0.70, 1.29)		0.66 (0.48, 0.91)*		0.93 (0.69, 1.25)	
NFL	Higher (n=669)	0.82 (0.61, 1.10)	0.622	0.81 (0.63, 1.05)	0.504	0.76 (0.62, 0.92)*	0.971
	Lower (n=1196)	0.92 (0.64, 1.31)		0.69 (0.44, 0.99)*		0.77 (0.58, 1.00)	
GFAP	Higher (n=647)	0.85 (0.62, 1.16)	0.884	0.76 (0.58, 1.01)	0.654	0.79 (0.63, 0.99)*	0.574
	Lower (n=1218)	0.87 (0.65, 1.18)		0.70 (0.52, 0.94)*		0.72 (0.56, 1.00)	

** p < 0.05 (nominally significant); ** p < (0.05/9) after Bonferroni correction; HR (hazard ratio); CI (confidence interval); p-int (p for two-way interaction); AMED = Alternate Mediterranean Diet Index; AHEI = Alternate Healthy Eating Index; rEDII = reversed Empirical Dietary Inflammatory Index; p-Tau217 = phosphorylated tau 217; NFL = neurofilament light; GFAP = glial fibrillary acidic protein*

Biomarker levels: p-tau217 (i) higher level: from 0.134 pg/mL to 6.92 pg/mL; (ii) lower level: from 0.00 pg/mL to 0.134 pg/mL; NFL (i) higher level: from 20.2 pg/mL to 389 pg/mL; (ii) lower level: from 3.06 to 20.2 pg/mL; GFAP (i) higher level: from 143 pg/mL to 6144 pg/mL; (ii) lower level: from 14.2 pg/mL to 142 pg/mL.

© 2026 Mrhar A et al. *JAMA Network Open*.

eTable 6. Sensitivity Analysis of the Association of Cumulative Diet Quality With 15-Year Dementia Due to Alzheimer Disease Risk, by Levels of Blood-Based Biomarkers of Alzheimer Disease-Related Pathology (p-tau217) and Nonspecific Neurodegeneration (NFL, GFAP) in a Competitive Risk Setting (n = 1865)

		Dietary pattern, HR (95% CI)					
		AMED		AHEI		rEDII	
			p-int		p-int		p-int
ptau217	Higher (n=589)	0.72 (0.52, 0.99)*	0.150	0.87 (0.68, 1.12)	0.280	0.75 (0.59, 0.95)*	0.102
	Lower (n=1276)	0.99 (0.72, 1.35)		0.70 (0.52, 0.95)*		0.94 (0.71, 1.24)	
NFL	Higher (n=669)	0.84 (0.62, 1.13)	0.698	0.84 (0.66, 1.08)	0.476	0.75 (0.56, 0.97)*	0.443
	Lower (n=1196)	0.92 (0.66, 1.28)		0.72 (0.51, 1.00)*		0.85 (0.69, 1.04)	
GFAP	Higher (n=647)	0.86 (0.64, 1.15)	0.810	0.82 (0.62, 1.07)	0.511	0.70 (0.54, 0.91)*	0.133
	Lower (n=1218)	0.91 (0.67, 1.22)		0.72 (0.55, 0.95)*		0.90 (0.72, 1.13)	

** p < 0.05 (nominally significant); ** p < (0.05/9) after Bonferroni correction; HR (hazard ratio); CI (confidence interval); p-int (p for two-way interaction); AMED = Alternate Mediterranean Diet Index; AHEI = Alternate Healthy Eating Index; rEDII = reversed Empirical Dietary Inflammatory Index; p-Tau217 = phosphorylated tau 217; NFL = neurofilament light; GFAP = glial fibrillary acidic protein*

Biomarker levels: p-tau217 (i) higher level: from 0.134 pg/mL to 6.92 pg/mL; (ii) lower level: from 0.00 pg/mL to 0.134 pg/mL; NFL (i) higher level: from 20.2 pg/mL to 389 pg/mL; (ii) lower level: from 3.06 to 20.2 pg/mL; GFAP (i) higher level: from 143 pg/mL to 6144 pg/mL; (ii) lower level: from 14.2 pg/mL to 142 pg/mL.

© 2026 Mrhar A et al. *JAMA Network Open*.

eTable 7. Ancillary Analysis Estimating the 10-Year Restricted Mean Time Lost at 10th and 90th Percentile of Average Diet Quality, by Levels of the Biomarkers of Alzheimer Disease-Related Pathology (p-tau217) and Nonspecific Neurodegeneration (NFL, GFAP)(n = 1865)

	Dietary pattern, 10 year RMTL difference (95% CI) by 10th and 90th precentile of adherence					
	AMED		AHEI		rEDII	
Higher level of:	10th	90th	10th	90th	10th	90th
ptau217 (n=589)	Ref.	-0.78 (-1.16, -0.40)*	Ref.	-0.66 (-1.03, -0.28)*	Ref.	-0.89 (-1.50, -0.29)*
NFL (n=669)	Ref.	-0.14 (-0.51, 0.24)	Ref.	-0.46 (-0.98, 0.16)	Ref.	-0.34 (-0.90, -0.18)*
GFAP (n=647)	Ref.	-0.15 (-0.52, 0.23)	Ref.	-0.35 (-0.73, 0.03)	Ref.	-0.53 (-0.91, -0.15)*

** $p < 0.05$ (nominally significant); ** $p < (0.05/9)$ after Bonferroni correction; HR (hazard ratio); CI (confidence interval); p-int (p for two-way interaction); AMED = Alternate Mediterranean Diet Index; AHEI = Alternate Healthy Eating Index; rEDII = reversed Empirical Dietary Inflammatory Index; p-Tau217 = phosphorylated tau 217; NFL = neurofilament light; GFAP = glial fibrillary acidic protein*

Biomarker levels: p-tau217: from 0.134 pg/mL to 6.92 pg/mL; NFL: from 20.2 pg/mL to 389 pg/mL; GFAP: from 143 pg/mL to 6144 pg/mL.

© 2026 Mrhar A et al. *JAMA Network Open*.

eTable 8. Ancillary Analysis Estimating the 10-Year Cumulative Incidence Function at 10th and 90th Percentile of Average Diet Quality, by Levels of the Biomarkers of Alzheimer Disease-Related Pathology (p-tau217) and Nonspecific Neurodegeneration (NFL, GFAP) (n = 1865)

	Dietary pattern, 10 year CIF difference (95% CI) by 10th and 90th precentile of adherence					
	AMED		AHEI		rEDII	
Higher level of:	10th	90th	10th	90th	10th	90th
Ptau217 (n=589)	Ref.	-0.18 (-0.33, -0.04)*	Ref.	-0.10 (-0.25, 0.04)	Ref.	-0.24 (-0.39, -0.09)*
NFL (n=669)	Ref.	-0.06 (-0.20, 0.09)	Ref.	-0.07 (-0.21, 0.07)	Ref.	-0.16 (-0.30, -0.01)*
GFAP (n=647)	Ref.	-0.07 (-0.21, 0.09)	Ref.	-0.13 (-0.23, 0.02)	Ref.	-0.17 (-0.31, -0.02)*

** p < 0.05 (nominally significant); ** p < (0.05/9) after Bonferroni correction; HR (hazard ratio); CI (confidence interval); p-int (p for two-way interaction); AMED = Alternate Mediterranean Diet Index; AHEI = Alternate Healthy Eating Index; rEDII = reversed Empirical Dietary Inflammatory Index; p-Tau217 = phosphorylated tau 217; NFL = neurofilament light; GFAP = glial fibrillary acidic protein*

Biomarker levels: p-tau217: from 0.134 pg/mL to 6.92 pg/mL; NFL: from 20.2 pg/mL to 389 pg/mL; GFAP: from 143 pg/mL to 6144 pg/mL.

© 2026 Mrhar A et al. *JAMA Network Open*.

eTable 9. Sensitivity Analysis of the Association of Cumulative Diet Quality With 15-Year Dementia Risk, by Blood-Based Biomarkers of Alzheimer Disease-Related Pathology (p-tau217) and Nonspecific Neurodegeneration (NFL, GFAP), Excluding Likely Misreported Dietary Data (n = 1846)

		Dietary pattern, HR (95% CI)					
		AMED		AHEI		EDII	
			p-int		p-int		p-int
ptau217	Higher (n=589)	0.81 (0.72, 1.13)	0.338	0.88 (0.70, 1.11)	0.049	0.69 (0.55, 0.84)**	0.011
	Lower (n=1276)	0.67 (0.59, 0.99)*		0.68 (0.54, 0.84)**		0.99 (0.80, 1.21)	
NFL	Higher (n=669)	0.91 (0.74, 1.16)	0.101	0.94 (0.78, 1.14)	0.009	0.79 (0.65, 0.94)*	0.601
	Lower (n=1196)	0.71 (0.54, 0.92)*		0.64 (0.48, 0.80)**		0.84 (0.67, 1.08)	
GFAP	Higher (n=647)	0.90 (0.73, 1.11)	0.304	0.80 (0.66, 1.00)*	0.832	0.71 (0.59, 0.88)*	0.132
	Lower (n=1218)	0.76 (0.59, 0.98)*		0.77 (0.62, 0.99)*		0.89 (0.72, 1.09)	

* p < 0.05 (nominally significant); ** p < (0.05/9) after Bonferroni correction; HR (hazard ratio); CI (confidence interval); p-int (p for two-way interaction); *AMED = Alternate Mediterranean Diet Index; AHEI = Alternate Healthy Eating Index; rEDII = reversed Empirical Dietary Inflammatory Index; p-Tau217 = phosphorylated tau 217*; NFL = neurofilament light; GFAP = glial fibrillary acidic protein

Biomarker levels: p-tau217 (i) higher level: from 0.134 pg/mL to 6.92 pg/mL; (ii) lower level: from 0.00 pg/mL to 0.134 pg/mL; NFL (i) higher level: from 20.2 pg/mL to 389 pg/mL; (ii) lower level: from 3.06 to 20.2 pg/mL; GFAP (i) higher level: from 143 pg/mL to 6144 pg/mL; (ii) lower level: from 14.2 pg/mL to 142 pg/mL.

Likely misreported dietary data was inspected as energy intake >5000 or <800 kcal/day in males and >4000 or <500 kcal/day in females (n=19) (8).

© 2026 Mrhar A et al. *JAMA Network Open*.

eTable 10. Sensitivity Analysis of the Association of Cumulative Diet Quality With 15-Year Dementia Risk, by Blood-Based Biomarkers of Alzheimer Disease-Related Pathology (p-tau217) and Nonspecific Neurodegeneration (NFL, GFAP), Excluding Participants With Cognitive Impairment, No Dementia (n = 1524)

		Dietary pattern, HR (95% CI)					
		AMED		AHEI		rEDII	
			p-int		p-int		p-int
ptau217	Higher (n=589)	0.98 (0.67, 1.34)	0.173	0.91 (0.67, 1.32)	0.023	0.68 (0.54, 0.86)**	0.027
	Lower (n=1276)	0.77 (0.57, 1.04)		0.68 (0.53, 0.86)**		1.01 (0.77, 1.38)	
NFL	Higher (n=669)	0.99 (0.74, 1.31)	0. 101	0.96 (0.76, 1.21)	0.039	0.83 (0.66, 0.99)*	0.645
	Lower (n=1196)	0.74 (0.54, 0.99)*		0.67 (0.51, 0.88)*		0.91 (0.67, 1.04)	
GFAP	Higher (n=647)	0.98 (0.75, 1.30)	0.361	0.87 (0.68, 1.11)	0.163	0.82 (0.63, 0.98)*	0.623
	Lower (n=1218)	0.82 (0.59, 1.13)		0.80 (0.60, 0.99)*		0.89 (0.69, 1.14)	

** p < 0.05 (nominally significant); ** p < (0.05/9) after Bonferroni correction*; HR (hazard ratio); CI (confidence interval); ref. (reference group); p-int (p for two-way interaction); *AMED = Alternate Mediterranean Diet Index; AHEI = Alternate Healthy Eating Index; rEDII = reversed Empirical Dietary Inflammatory Index;* p-Tau217 = phosphorylated tau 217; NFL = neurofilament light; GFAP = glial fibrillary acidic protein

Biomarker levels: p-tau217 (i) higher level: from 0.134 pg/mL to 6.92 pg/mL; (ii) lower level: from 0.00 pg/mL to 0.134 pg/mL; NFL (i) higher level: from 20.2 pg/mL to 389 pg/mL; (ii) lower level: from 3.06 to 20.2 pg/mL; GFAP (i) higher level: from 143 pg/mL to 6144 pg/mL; (ii) lower level: from 14.2 pg/mL to 142 pg/mL.

© 2026 Mrhar A et al. *JAMA Network Open*.

eTable 11. Sensitivity Analysis of the Association of Cumulative Diet Quality With 15-Year Dementia Risk, by Blood-Based Biomarkers of Alzheimer Disease-Related Pathology (p-tau217) and Nonspecific Neurodegeneration (NFL, GFAP), Excluding Participants Who Developed Dementia During the First and Second Follow-Up (n = 1783)

		Dietary pattern, HR (95% CI)					
		AMED		AHEI		EDII	
			p-int		p-int		p-int
ptau217	Higher (n=649)	0.88 (0.70, 1.10)	0.457	0.93 (0.76, 1.16)	0.045	0.70 (0.57, 0.88)*	0.037
	Lower (n=1216)	0.77 (0.61, 0.99)*		0.70 (0.57, 0.87)**		0.97 (0.79, 1.21)	
NFL	Higher (n=648)	0.91 (0.73, 1.13)	0. 251	0.95 (0.79, 1.15)	0.022	0.77 (0.64, 0.93)*	0.260
	Lower (n=1217)	0.75 (0.58, 0.97)*		0.68 (0.54, 0.86)**		0.91 (0.72, 1.14)	
GFAP	Higher (n=638)	0.91 (0.67, 1.00)	0.214	0.84 (0.69, 1.02)	0.559	0.72 (0.59, 0.88)*	0.084
	Lower (n=1227)	0.69 (0.53, 0.90)*		0.82 (0.64, 0.99)*		0.91 (0.75, 1.12)	

* p < 0.05 (nominally significant); ** p < (0.05/9) after Bonferroni correction; HR (hazard ratio); CI (confidence interval); p-int (p for two-way interaction); *AMED = Alternate Mediterranean Diet Index; AHEI = Alternate Healthy Eating Index; rEDII = reversed Empirical Dietary Inflammatory Index;* p-Tau217 = phosphorylated tau 217; NFL = neurofilament light; GFAP = glial fibrillary acidic protein

Biomarker levels: p-tau217 (i) higher level: from 0.134 pg/mL to 6.92 pg/mL; (ii) lower level: from 0.00 pg/mL to 0.134 pg/mL; NFL (i) higher level: from 20.2 pg/mL to 389 pg/mL; (ii) lower level: from 3.06 to 20.2 pg/mL; GFAP (i) higher level: from 143 pg/mL to 6144 pg/mL; (ii) lower level: from 14.2 pg/mL to 142 pg/mL.

© 2026 Mrhar A et al. *JAMA Network Open*.

eTable 12. Sensitivity Analysis of the Association of Cumulative Diet Quality With 15-Year Dementia Risk, by Blood-Based Biomarkers of Alzheimer Disease-Related Pathology (p-tau217) and Nonspecific Neurodegeneration (NFL, GFAP), Using Highest Tertile of Biomarkers as a Cutoff (n = 1865)

		Dietary pattern, HR (95% CI)					
		AMED		AHEI		EDII	
			p-int		p-int		p-int
ptau217	Higher (n=589)	0.98 (0.71, 1.36)	0.285	0.89 (0.69, 1.16)	0.363	0.58 (0.45, 0.76)**	0.016
	Lower (n=1276)	0.76 (0.53, 1.09)		0.74 (0.54, 1.00)*		0.98 (0.71, 1.35)	
NFL	Higher (n=669)	0.96 (0.65, 1.40)	0. 749	0.85 (0.66, 1.09)	0.687	0.75 (0.61, 0,93)*	0.759
	Lower (n=1196)	0.80 (0.58, 1.11)		0.78 (0.53, 1.13)		0.80 (0.57, 1.10)	
GFAP	Higher (n=647)	0.89 (0.65, 1.23)	0.844	0.81 (0.61, 1.08)	0.348	0.76 (0.59, 0.97)*	0.910
	Lower (n=1218)	0.86 (0.61, 1.22)		0.78 (0.54, 0.99)*		0.77 (0.60, 1.01)	

* p < 0.05 (nominally significant); ** p < (0.05/9) after Bonferroni correction; HR (hazard ratio); CI (confidence interval); p-int (p for two-way interaction); *AMED = Alternate Mediterranean Diet Index; AHEI = Alternate Healthy Eating Index; rEDII = reversed Empirical Dietary Inflammatory Index;* p-Tau217 = phosphorylated tau 217; NFL = neurofilament light; GFAP = glial fibrillary acidic protein

Biomarker levels: p-tau217 (i) higher level: from 0.134 pg/mL to 6.92 pg/mL; (ii) lower level: from 0.00 pg/mL to 0.134 pg/mL; NFL (i) higher level: from 20.2 pg/mL to 389 pg/mL; (ii) lower level: from 3.06 to 20.2 pg/mL; GFAP (i) higher level: from 143 pg/mL to 6144 pg/mL; (ii) lower level: from 14.2 pg/mL to 142 pg/mL.

© 2026 Mrhar A et al. *JAMA Network Open*.

eTable 13. Sensitivity Analysis of the Association of Cumulative Adherence to the Reversed Dietary Inflammatory Index With 15-Year Dementia Risk, Stratified by Blood-Based Biomarkers of Alzheimer Disease-Related Pathology (p-tau217) and Nonspecific Neurodegeneration (NFL, GFAP) (n = 1865)

		Dietary pattern, HR (95% CI)<br>rDII	p-int
ptau217	Higher (n=589)	0.75 (0.56, 0.99)*	0.048
	Lower (n=1276)	0.95 (0.83, 1.15)	
NFL	Higher (n=669)	0.72 (0.54, 0.95)*	0.363
	Lower (n=1196)	0.83 (0.64, 1.06)	
GFAP	Higher (n=647)	0.73 (0.54, 0.98)*	0.240
	Lower (n=1218)	0.87 (0.69, 1.09)	

* p < 0.05 (nominally significant); ** p < (0.05/9) after Bonferroni correction; HR (hazard ratio); CI (confidence interval); p-int (p for two-way interaction); *AMED = Alternate Mediterranean Diet Index; AHEI = Alternate Healthy Eating Index; rEDII = reversed Empirical Dietary Inflammatory Index;* p-Tau217 = phosphorylated tau 217; NFL = neurofilament light; GFAP = glial fibrillary acidic protein

Biomarker levels: p-tau217 (i) higher level: from 0.134 pg/mL to 6.92 pg/mL; (ii) lower level: from 0.00 pg/mL to 0.134 pg/mL; NFL (i) higher level: from 20.2 pg/mL to 389 pg/mL; (ii) lower level: from 3.06 to 20.2 pg/mL; GFAP (i) higher level: from 143 pg/mL to 6144 pg/mL; (ii) lower level: from 14.2 pg/mL to 142 pg/mL.

© 2026 Mrhar A et al. *JAMA Network Open*.

eTable 14. Subgroup Analysis of the Association of Cumulative Diet Quality With 15-Year Dementia Onset, by Levels of Blood-Based Biomarkers of Alzheimer Disease-Related Pathology (p-tau217), Nonspecific Neurodegeneration (NFL, GFAP), and Age (n = 1865)

		Dietary pattern, HR (95% CI)								
		AMED			AHEI			rEDII		
		< 78 years	≥ 78 years	p-int	< 78 years	≥ 78 years	p-int	< 78 years	≥ 78 years	p-int
ptau217	Higher (n=649)	0.88 (0.59, 1.32)	0.74 (0.57, 0.96)*	0.122	1.01 (0.74, 1.57)	0.72 (0.56, 0.92)*	0.021	0.73 (0.48, 1.11)	0.63 (0.52, 0.77)**	0.790
	Lower (n=1216)	0.58 (0.40, 0.84)*	0.81 (0.60, 1.10)		0.52 (0.38, 0.71)**	0.76 (0.58, 0.99)*		1.00 (0.70, 1.45)	0.95 (0.70, 1.29)	
NFL	Higher (n=648)	0.74 (0.47, 1.18)	0.83 (0.66, 1.05)	0.795	0.87 (0.57, 1.33)	0.87 (0.71, 1.06)	0.765	0.82 (0.56, 1.20)	0.74 (0.63, 0.88)*	0.717
	Lower (n=1217)	0.66 (0.46, 0.94)*	0.67 (0.46, 0.98)*		0.60 (0.44, 0.81)	0.53 (0.34, 0.82)		0.90 (0.66, 1.23)	0.72 (0.47, 1.12)	
GFAP	Higher (n=638)	0.77 (0.50, 1.17)	0.83 (0.66, 1.04)	0.911	0.61 (0.40, 0.92)*	0.81 (0.66, 0.99)*	0.186	0.75 (0.52, 1.07)	0.74 (0.61, 0.89)*	0.346
	Lower (n=1227)	0.66 (0.45, 0.98)*	0.69 (0.48, 0.98)*		0.73 (0.53, 1.02)	0.62 (0.42, 0.91)*		1.00 (0.71, 1.41)	0.73 (0.51, 1.04)	

** p < 0.05 (nominally significant); ** p < (0.05/27) after Bonferroni correction; HR (hazard ratio); CI (confidence interval); p-int (p for three-way interaction); AMED = Alternate Mediterranean Diet Index; AHEI = Alternate Healthy Eating Index; rEDII = reversed Empirical Dietary Inflammatory Index;* p-Tau217 = phosphorylated tau 217; *NFL = neurofilament light; GFAP = glial fibrillary acidic protein*

Biomarker levels: p-tau217 (i) higher level: from 0.134 pg/mL to 6.92 pg/mL; (ii) lower level: from 0.00 pg/mL to 0.134 pg/mL; NFL (i) higher level: from 20.2 pg/mL to 389 pg/mL; (ii) lower level: from 3.06 to 20.2 pg/mL; GFAP (i) higher level: from 143 pg/mL to 6144 pg/mL; (ii) lower level: from 14.2 pg/mL to 142 pg/mL.

© 2026 Mrhar A et al. *JAMA Network Open*.

eTable 15. Subgroup Analysis of the Association of Cumulative Diet Quality With 15-Year Dementia Onset, by Levels of Blood-Based Biomarkers of Alzheimer Disease-Related Pathology (p-tau217), and Nonspecific Neurodegeneration (NFL, GFAP), and *APOE*-e4 (n = 1821)

		Dietary pattern, HR (95% CI)								
		AMED			AHEI			EDII		
		no APOE-e4	APOE-e4	p-int	no APOE-e4	APOE-e4	p-int	no APOE-e4	APOE-e4	p-int
ptau217	Higher (n=649)	0.88 (0.65, 1.20)	0.67 (0.49, 0.91)*	0.699	0.90 (0.67, 1.21)	0.67 (0.49, 0.91)	0.707	0.59 (0.48, 0.73)**	0.74 (0.54, 1.01)	0.303
	Lower (n=1216)	0.83 (0.62, 1.11)	0.55 (0.37, 0.82)*		0.69 (0.54, 0.88)*	0.57 (0.39, 0.84)		0.79 (0.64, 0.99)*	1.35 (0.92, 1.98)	
NFL	Higher (n=648)	0.94 (0.73, 1.22)	0.64 (0.46, 0.88)*	0.608	0.91 (0.72, 1.14)	0.76 (0.57, 1.01)	0.632	0.73 (0.60, 0.87)*	0.81 (0.62, 1.04)	0.431
	Lower (n=1217)	0.74 (0.48, 1.13)	0.60 (0.42, 0.85)*		0.66 (0.46, 0.97)*	0.47 (0.32, 0.69)*		0.71 (0.56, 0.91)	0.99 (0.66, 1.49)	
GFAP	Higher (n=638)	1.01 (0.76, 1.33)	0.66 (0.50, 0.87)*	0.454	0.93 (0.72, 1.21)	0.61 (0.47, 0.80)	0.325	0.71 (0.57, 0.88)*	0.74 (0.57, 0.95)*	0.024
	Lower (n=1227)	0.71 (0,51, 1.00)*	0.60 (0.39, 0.93)*		0.68 (0.50, 0.94)*	0.63 (0.41, 0.97)*		0.70 (0.57, 0.86)*	1.43 (0.91, 2.27)	

* p < 0.05 (nominally significant); ** p < (0.05/27) after Bonferroni correction; HR (hazard ratio); CI (confidence interval); p-int (p for three-way interaction); AMED = Alternate Mediterranean Diet Index; AHEI = Alternate Healthy Eating Index; rEDII = reversed Empirical Dietary Inflammatory Index; p-Tau217 = phosphorylated tau 217; NFL = neurofilament light; GFAP = glial fibrillary acidic protein

Biomarker levels: p-tau217 (i) higher level: from 0.134 pg/mL to 6.92 pg/mL; (ii) lower level: from 0.00 pg/mL to 0.134 pg/mL; NFL (i) higher level: from 20.2 pg/mL to 389 pg/mL; (ii) lower level: from 3.06 to 20.2 pg/mL; GFAP (i) higher level: from 143 pg/mL to 6144 pg/mL; (ii) lower level: from 14.2 pg/mL to 142 pg/mL.

© 2026 Mrhar A et al. *JAMA Network Open*.

eTable 16. Subgroup Analysis of the Association of Cumulative Diet Quality With 15-Year Dementia Onset, by Levels of Blood-Based Biomarkers of Alzheimer Disease-Related Pathology (p-tau217), Nonspecific Neurodegeneration (NFL, GFAP), and Sex (n = 1865)

		Dietary pattern, HR (95% CI)								
		AMED			AHEI			EDII		
		Males	Females	p-int	Males	Females	p-int	Males	Females	p-int
ptau217	Higher (n=649)	0.68 (0.48, 0.96)*	0.87 (0.66, 1.16)	0.456	0.75 (0.54, 1.06)	0.84 (0.63, 1.11)	0.955	0.58 (0.45, 0.76)**	0.68 (0.53, 0.87)**	0.185
	Lower (n=1216)	0.73 (0.46, 1.16)	0.72 (0.56, 0.94)*		0.63 (0.45, 0.90)*	0.69 (0.53, 0.89)*		1.01 (0.74, 1.60)	0.88 (0.67, 1.14)	
NFL	Higher (n=648)	0.61 (0.42, 0.88)**	0.97 (0.76, 1.12)	0. 040	0.72 (0.52, 0.98)*	0.96 (0.77, 1.21)	0.458	0.65 (0.51, 0.83)**	0.79 (0.65, 0.96)*	0.234
	Lower (n=1217)	0.78 (0.51, 1.21)	0.61 (0.44, 0.85)*		0.60 (0.42, 0.86)**	0.56 (0.39, 0.80)*		0.91 (0.58, 1.43)	0.77 (0.58, 1.01)	
GFAP	Higher (n=638)	0.70 (0.47, 1.03)	0.89 (0.71, 1.12)	0.493	0.68 (0.45, 0.96)*	0.79 (0.63, 0.99)*	0.929	0.78 (0.54, 1.12)	0.69 (0.56, 0.85)**	0.251
	Lower (n=1227)	0.69 (0.46, 1.01)	0.69 (0.49, 0.97)*		0.76 (0.53, 1.09)	0.70 (0.50, 0.98)*		0.73 (0.52, 1.02)	0.91 (0.69, 1.21)	

* p < 0.05 (nominally significant); ** p < (0.05/27) after Bonferroni correction; HR (hazard ratio); CI (confidence interval); p-int (p for three-way interaction); p-Tau217 = phosphorylated tau 217; NFL = neurofilament light; GFAP = glial fibrillary acidic protein

Bi Biomarker levels: p-tau217 (i) higher level: from 0.134 pg/mL to 6.92 pg/mL; (ii) lower level: from 0.00 pg/mL to 0.134 pg/mL; NFL (i) higher level: from 20.2 pg/mL to 389 pg/mL; (ii) lower level: from 3.06 to 20.2 pg/mL; GFAP (i) higher level: from 143 pg/mL to 6144 pg/mL; (ii) lower level: from 14.2 pg/mL to 142 pg/mL.

© 2026 Mrhar A et al. *JAMA Network Open*.

eFigure 1. Flow Diagram of Study Sample Selection

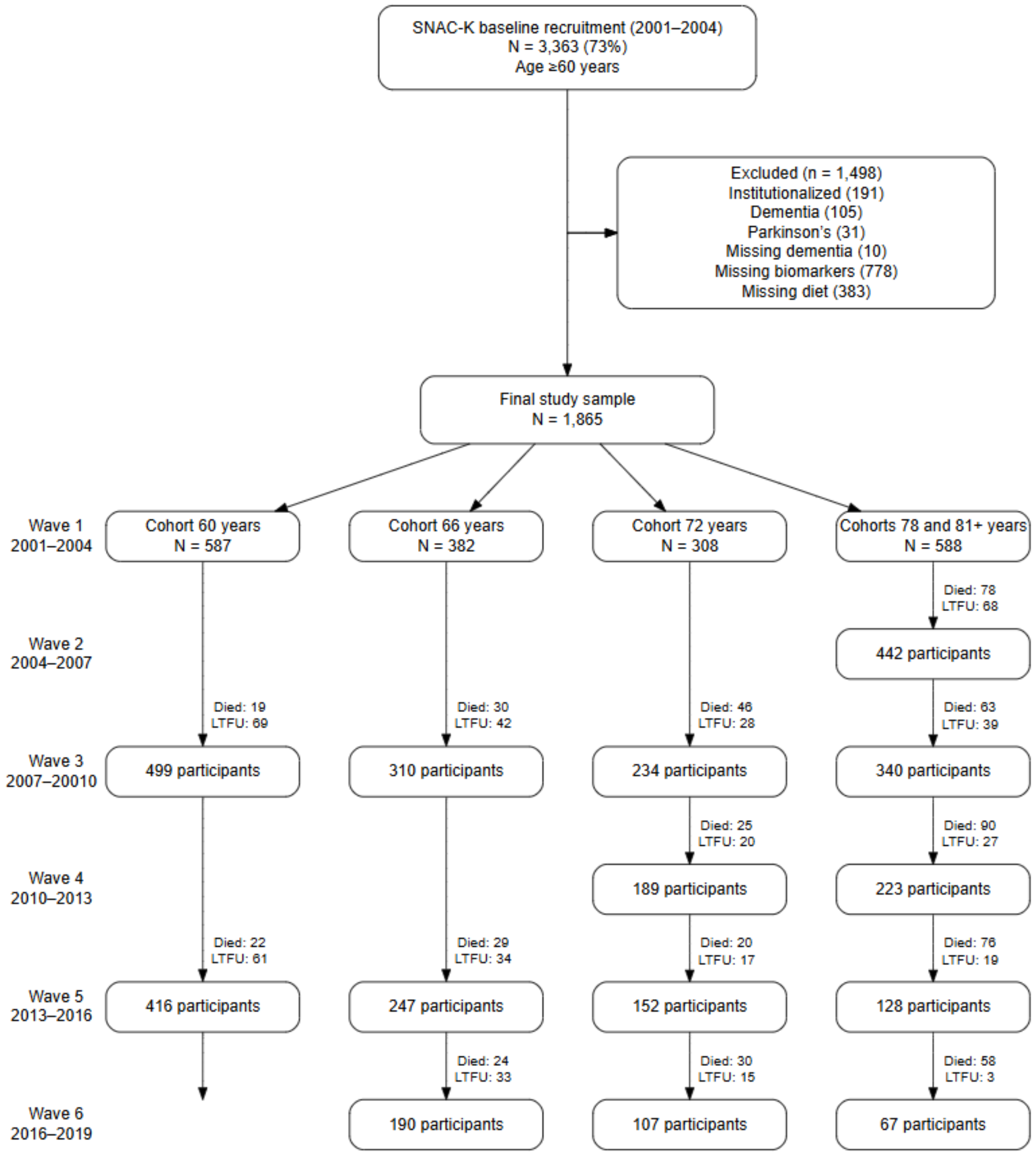

SNAC-K = Aging and Care in Kungsholmen (SNAC-K) Cohort; LTFU = Lost to follow-up

© 2026 Mrhar A et al. *JAMA Network Open*.

eFigure 2. Overlap of High Levels of Blood-Based Biomarkers of Alzheimer Disease-Related Pathology (p-tau217) and Nonspecific Neurodegeneration (NFL, GFAP) (n = 1865)

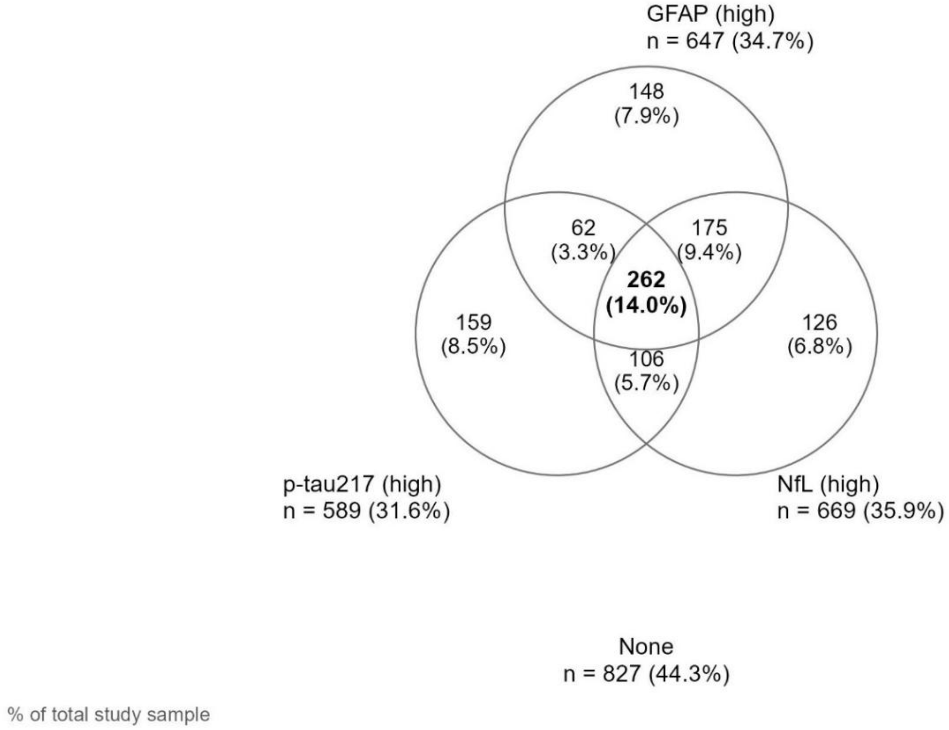

p-tau217 = phosphorylated tau 217; NFL = neurofilament light; GFAP = glial fibrillary acidic protein

Biomarker levels: p-tau217 (i) higher level: from 0.134 pg/mL to 6.92 pg/mL; (ii) lower level: from 0.00 pg/mL to 0.134 pg/mL; NFL (i) higher level: from 20.2 pg/mL to 389 pg/mL; (ii) lower level: from 3.06 to 20.2 pg/mL; GFAP (i) higher level: from 143 pg/mL to 6144 pg/mL; (ii) lower level: from 14.2 pg/mL to 142 pg/mL.

© 2026 Mrhar A et al. *JAMA Network Open*.

eFigure 3. Spline Associations of Cumulative Adherence to Alternate Mediterranean Diet (AMED) With 15-Year Dementia Risk, Stratified by Blood-Based Biomarkers of Alzheimer Disease-Related Pathology (p-tau217) and Nonspecific Neurodegeneration (NFL, GFAP) (n=1865)

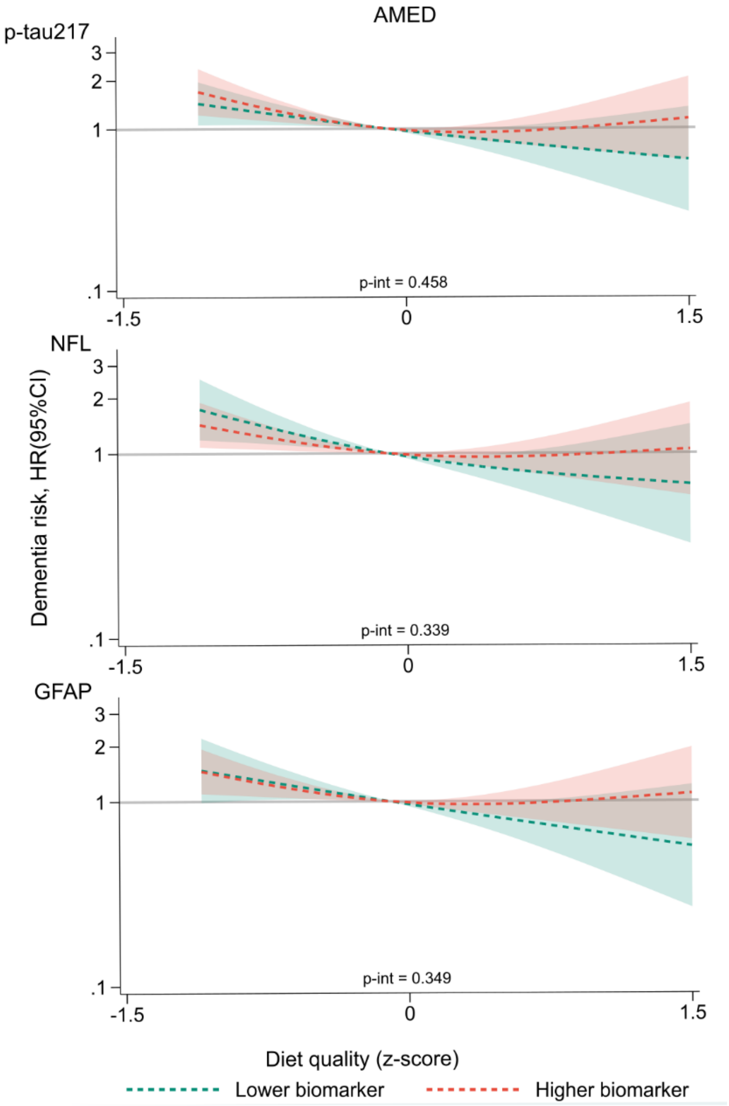

p-Tau217 = phosphorylated tau 217; NFL = neurofilament light; GFAP = glial fibrillary acidic protein.

Biomarker levels: p-tau217 (i) higher level: from 0.134 pg/mL to 6.92 pg/mL; (ii) lower level: from 0.00 pg/mL to 0.134 pg/mL; NFL (i) higher level: from 20.2 pg/mL to 389 pg/mL; (ii) lower level: from 3.06 to 20.2 pg/mL; GFAP (i) higher level: from 143 pg/mL to 6144 pg/mL; (ii) lower level: from 14.2 pg/mL to 142 pg/mL.

Results were obtained from Cox proportional hazards regression models. Diet indices were standardized and modeled as 3-knot restricted cubic splines. Hazard ratios (HR) and 95% confidence intervals (CI) were plotted for adherence to the dietary patterns above the 1st percentile and below the 99th percentile, and obtained from models with interaction terms between the dietary pattern and biomarker level. Models were adjusted for (1) sociodemographic variables: sex, age, longest held occupation (manual worker or not), living arrangement (living alone or not), and highest educational level (elementary school, high school, or university); (2) lifestyle variables: smoking status (have never smoked, former smoker, or current smoker), light and moderate physical activity level (never, less than

© 2026 Mrhar A et al. *JAMA Network Open*.

monthly, monthly, weekly, or daily), body mass index (BMI) (kg/m^2), and energy intake (kcal/day); and (3) morbidity: diabetes, heart diseases (atrial fibrillation, heart failure, ischemic heart disease, or heart valve disease), cerebrovascular disease, depression and mood diseases, hypertension, anemia, and chronic kidney disease.

© 2026 Mrhar A et al. *JAMA Network Open*.

eFigure 4. Cumulative Incidence of Dementia, Stratified by Diet Quality and Levels of Blood-Based Biomarkers of Alzheimer Disease-Related Pathology (p-tau217) and Nonspecific Neurodegeneration (NFL, GFAP) (solid lines)and 10-Year Restricted Mean Time Lost Difference Between Lower and Higher Diet Quality Among the Participants With Higher Levels of the Biomarkers (gray shading) (n = 1865)

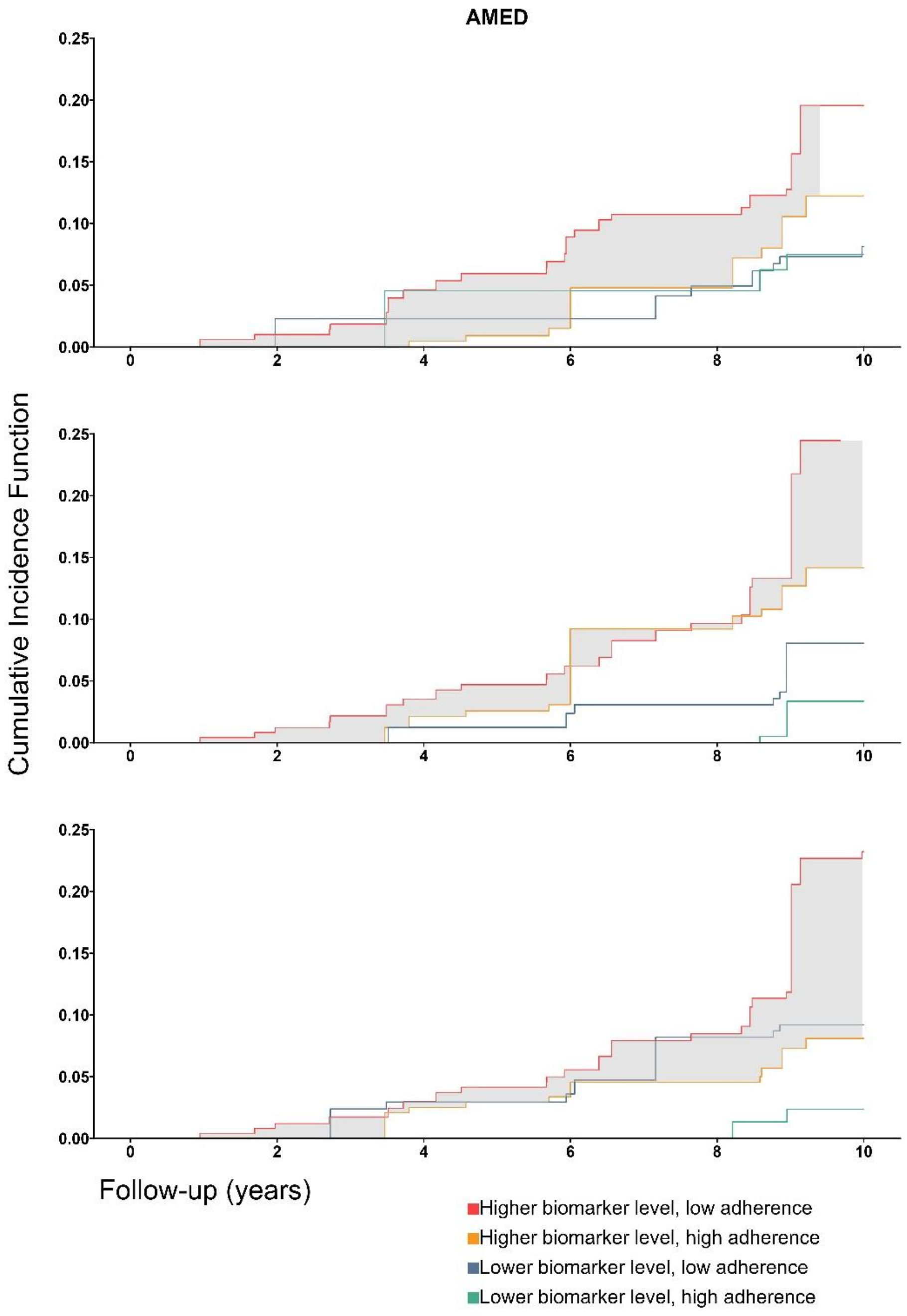

AMED = Alternative Mediterranean Diet Index, p-Tau217 = phosphorylated tau 217; NFL = neurofilament light; GFAP = glial fibrillary acidic protein

Biomarker levels: p-tau217 (i) higher level: from 0.134 pg/mL to 6.922 pg/mL; (ii) lower level: from 0.000 pg/mL to 0.134 pg/mL; NFL (i) higher level: from 20.18 pg/mL to 389.05; (ii) lower level: from 3.06 to 20.17 pg/mL; GFAP (i) higher level: from 142.6 pg/mL to 6143.9 pg/mL; (ii) lower level: from 14.2 pg/mL to 142.1 pg/mL.

© 2026 Mrhar A et al. *JAMA Network Open*.

Results were obtained from the Aalen-Johansen estimator, accounting for the competing risk of death and adjusted for (1) sociodemographic variables: sex, age, longest held occupation (manual worker or not), living arrangement (living alone or not), and highest educational level (elementary school, high school, or university); (2) lifestyle variables: smoking status (have never smoked, former smoker, or current smoker), light and moderate physical activity level (never, less than monthly, monthly, weekly, or daily), body mass index (BMI) (kg/m^2), and energy intake (kcal/day); and (3) morbidity: diabetes, heart diseases (atrial fibrillation, heart failure, ischemic heart disease, or heart valve disease), cerebrovascular disease, depression and mood diseases, hypertension, anemia, and chronic kidney disease.

© 2026 Mrhar A et al. *JAMA Network Open*.

eReferences

1. Fung TT, Rexrode KM, Mantzoros CS, Manson JE, Willett WC, Hu FB. Mediterranean diet and incidence of and mortality from coronary heart disease and stroke in women. Circulation. 2009 Mar 3;119(8):1093–100. doi:10.1161/CIRCULATIONAHA.108.816736 PubMed PMID: 19221219.

2. Chiuve SE, Fung TT, Rimm EB, Hu FB, McCullough ML, Wang M, et al. Alternative dietary indices both strongly predict risk of chronic disease. Journal of Nutrition. 2012 Jun 1;142(6):1009–18. doi:10.3945/jn.111.157222 PubMed PMID: 22513989.

3. Varraso R, Chiuve SE, Fung TT, Barr RG, Hu FB, Willett WC, et al. Alternate Healthy Eating Index 2010 and risk of chronic obstructive pulmonary disease among US women and men: Prospective study. BMJ (Online). 2015 Feb 3;350. doi:10.1136/bmj.h286 PubMed PMID: 25649042.

4. Al-Ibrahim AA, Jackson RT. Healthy eating index versus alternate healthy index in relation to diabetes status and health markers in U.S. adults: NHANES 2007-2010. Nutr J. 2019 Apr 17;18(1). doi:10.1186/s12937-019-0450-6 PubMed PMID: 30995902.

5. Tabung FK, Smith-Warner SA, Chavarro JE, Wu K, Fuchs CS, Hu FB, et al. Development and validation of an empirical dietary inflammatory index. Journal of Nutrition. 2016 Aug 1;146(8):1560–70. doi:10.3945/jn.115.228718 PubMed PMID: 27358416.

6. Andersen PK, Pohar Perme M. Pseudo-observations in survival analysis. Statistical Methods in Medical Research. 2010. p. 71–99. doi:10.1177/0962280209105020 PubMed PMID: 19654170.

7. Parner ET, Andersen PK, Overgaard M. Regression models for censored time-to-event data using infinitesimal jack-knife pseudo-observations, with applications to left-truncation. Lifetime Data Anal. 2023 Jul 1;29(3):654–71. doi:10.1007/s10985-023-09597-5 PubMed PMID: 37157038.

8. Willett W. Nutritional Epidemiology. 3rd ed. Vol. 40. Oxford University Press; 2013.

9. Shivappa N, Steck SE, Hurley TG, Hussey JR, Hébert JR. Designing and developing a literature-derived, population-based dietary inflammatory index. Public Health Nutr. 2014;17(8):1689–96. doi:10.1017/S1368980013002115 PubMed PMID: 23941862.

© 2026 Mrhar A et al. *JAMA Network Open*.
