# Supplementary material for: Diet Quality and Dementia Risk in Older Adults With Alzheimer Pathology
Source: JAMA Netw Open. 2026 Jun 25;9(6):e2620254. doi: 10.1001/jamanetworkopen.2026.20254 (PMC13306305; doi:10.1001/jamanetworkopen.2026.20254)
Supplement: Supplement 2. — Data Sharing Statement [file jamanetwopen-e2620254-s002.pdf]

**Data Sharing Statement**

**Data**
**Data available:** Yes
**Data types:** Deidentified participant data
**How to access data:** Data are from the SNAC-K project, a population-based study on aging and dementia (http://www.snac-k.se/). Access to these original data is available to the research community upon approval by the SNAC-K organization. Applications for accessing these data can be submitted through http://www.snac-k.se/.
**When available:** With publication

**Supporting Documents**
**Document types:** None

**Additional Information**
**Who can access the data:** Access to these original data is available to the research community upon approval by the SNAC-K organization. Applications for accessing these data can be submitted through http://www.snac-k.se/.
**Types of analyses:** Analyses approved by the SNAC-K organization upon application.
**Mechanisms of data availability:** After approval of a proposal.
